# Balancing Donor‐Acceptor and Dispersion Effects in Heavy Main Group Element π Interactions: Effect of Substituents on the Pnictogen⋅⋅⋅π Arene Interaction

**DOI:** 10.1002/cphc.201900747

**Published:** 2019-09-12

**Authors:** Małgorzata Krasowska, Ana‐Maria Fritzsche, Michael Mehring, Alexander A. Auer

**Affiliations:** ^1^ Max-Planck-Institut für Kohlenforschung Kaiser-Wilhelm-Platz 1 45470 Mülheim an der Ruhr Germany; ^2^ Technische Universität Chemnitz Straße der Nationen 62 09107 Chemnitz Germany

**Keywords:** ab initio calculations, computational chemistry, intermolecular interactions, organometallic chemistry, pnictogen compounds

## Abstract

High‐level ab initio calculations using the DLPNO‐CCSD(T) method in conjunction with the local energy decomposition (LED) were performed to investigate the nature of the intermolecular interaction in bismuth trichloride adducts with π arene systems. Special emphasis was put on the effect of substituents in the aromatic ring. For this purpose, benzene derivatives with one or three substituents (R=NO_2_, CF_3_, OCHO, OH, and NH_2_) were chosen and their influence on donor‐acceptor interaction as well as on the overall interaction strength was examined. Local energy decomposition was performed to gain deeper insight into the composition of the interaction. Additionally, the study was extended to the intermolecular adducts of arsenic and antimony trichloride with benzene derivatives having one substituent (R=NO_2_ and NH_2_) in order to rationalize trends in the periodic table. The analysis of natural charges and frontier molecular orbitals shows that donor‐acceptor interactions are of π→σ* type and that their strength correlates with charge transfer and orbital energy differences. An analysis of different bonding motifs (Bi⋅⋅⋅π arene, Bi⋅⋅⋅R, and Cl⋅⋅⋅π arene) shows that if dispersion and donor‐acceptor interaction coincide as the donor highest occupied molecular orbital (HOMO) of the arene is delocalized over the π system, the M⋅⋅⋅π arene motif is preferred. If the donor HOMO is localized on the substituent, R⋅⋅⋅π arene bonding motifs are preferred. The Cl⋅⋅⋅π arene bonding motif is the least favorable with the lowest overall interaction energy.

## Graphical abstract

**Balanced interactions**: High level ab initio calculations show how the delicate balance between the dominating dispersion contribution and the directional donor‐acceptor component in pnictogen⋅⋅⋅π interactions leads to different structural motifs depending on the substitution pattern in the arene.

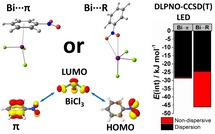

## 1 Introduction

The chemistry of intermolecular adducts of heavy pnictogen(III) compounds has been subject of study since the late 19^th^ century.^1^ Despite this early recognition the interest in this type of compounds was rather low maybe because applications were not expected, but re‐emerged with the growing interest in weak interactions as important force in supramolecular chemistry.^2^, ^3^, ^4^, ^5^, ^6^, ^7^, ^8^, ^9^ Furthermore, such weak interactions might also play a crucial role in some biological systems as discussed by Frontera and coworkers.^9^ In addition to numerous examples showing intermolecular pnicogen⋅⋅⋅π arene interaction, systems with intramolecular interaction were less frequently reported, especially for bismuth.^10^, ^11^, ^12^, ^13^, ^14^, ^15^, ^16^, ^17^, ^18^ However, these instances demonstrate that this rather weak interaction enables stabilization of unusual compounds and might support catalytic processes.^14^

In order to rationalize what determines molecular and crystal structures, understanding the basic components of the pnictogen⋅⋅⋅π arene interaction is essential. Compounds containing heavy main group elements are especially interesting as they can act as dispersion energy donors (DED) on the one hand, but their molecular structure can be modified by introducing various substituents, hence introducing donor‐acceptor properties on the other hand. This interplay was underrepresented in most of the previous studies, although Frontera and coworkers demonstrated that electron rich ligands strengthen and electron poor ligands weaken the intermolecular interactions in pnictogen⋅⋅⋅π arene adducts.^9^

Our previous studies^8^, ^19^, ^20^, ^21^ focused on different dispersion adducts of aromatic systems with trivalent heavy pnictogen compounds of the form MX_3_. In our initial publication,^8^ the interaction between bismuth(III) compounds BiX_3_ (where X=H, CH_3_, OH, OCH_3_, F, Cl, Br) and benzene was studied using density functional theory (DFT) and perturbation theory (MP2) where only MP2 yielded qualitatively correct results. It was concluded that the interaction in intermolecular adducts of Bi(CH_3_)_3_ with benzene is purely dispersive in nature, while the interaction in BiX_3_ (X=halogen) adducts is an interplay between dispersion and donor‐acceptor properties arising from the π(benzene)→σ*(BiX_3_) charge transfer. The magnitude of the charge transfer depends on the electron acceptor properties determined by the substituents on bismuth. In a recent publication^20^ we extended the study to other heavy pnictogen compounds of the type MX_3_ where M=As, Sb, Bi and X=CH_3_, OCH_3_, Cl with a focus on the electronic structure of the pnictogen⋅⋅⋅π arene interaction. Methods like dispersion‐corrected density functional theory (DFT‐D3) and the domain‐based localized pair natural orbitals coupled cluster approximation^22^, ^23^, ^24^, ^25^, ^26^, ^27^, ^28^ [DLPNO‐CCSD(T)] were used to investigate the pnictogen⋅⋅⋅π arene interaction. The DLPNO‐CC approximation offers qualitatively accurate results for large molecular systems and the interaction energy obtained at the DLPNO‐CC level of theory can be further decomposed into physically meaningful contributions by means of local energy decomposition^29^, ^30^ (LED). We found that the interaction energies calculated at the DLPNO‐CCSD(T) level increase from arsenic to antimony to bismuth and range from −10 kJ mol^−1^ to −40 kJ mol^−1^ depending on the pnictogen and substituent, which is in accordance with increasing polarizability of the specific pnictogen compound. Different substituents X on the pnictogen atom modulate the character of the interaction, with the interaction of benzene with trimethylpnictogen compounds being purely dispersive. On the other hand, the interaction in the pnictogen trichloride adducts is a mixture of dispersion and donor‐acceptor interaction. The donor‐acceptor interaction, just as the interaction energies, increases following the order arsenic<antimony<bismuth.

All previous investigations focused mainly on the role of the heavy pnictogen compound and how its electronic structure influences the nature of intermolecular interactions and its strength. However, most of the examples of intermolecular and intramolecular adducts between pnictogen compounds and aromatic molecules as described in the literature differ mainly in the substitution pattern of the aromatic systems.^1^, ^5^, ^6^

The influence of the aromatic substituent on the pnictogen⋅⋅⋅π arene interaction in PH_2_Cl adducts with various benzene derivatives with one substituent (R=H, OH, NH_2_, CH_3_, Br, Cl, F, CN, NO_2_) was investigated recently using computational chemistry methods.^31^ In accordance with the results of the Frontera group,^9^ the MP2 study revealed that electron withdrawing groups weaken binding affinity while electron donating substituents increase it and that charge transfer plays an important role in the intermolecular interaction. In this study, we examine if a substituent introduced at the aromatic ring influences the interaction in heavy pnictogen adducts with substituted arenes. For this purpose, we focused on bismuth trichloride adducts with various substituted benzenes owing to the fact that the interaction in the BiCl_3_⋅⋅⋅benzene adduct is a combination of dispersion and donor‐acceptor interactions. Regarding the full range of possible intramolecular interactions of heavy main group element compounds we have also included further interaction motifs in our investigations. We aim at quantifying and rationalizing the underlying effects that lead to the formation of certain structural motifs in molecular assemblies, adducts and solids.

**Scheme 1:**
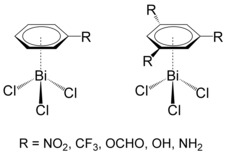
Schematic representation of studied systems.

In the first part of this paper, interaction potentials for the idealized structures of BiCl_3_⋅⋅⋅π arene adducts (see Scheme 1) with substituted benzenes computed using the DLPNO‐CCSD(T) method are discussed. Here, we investigate in detail how substitution influences the nature of the interaction and its strength. Subsequently, interaction potentials for arsenic and antimony with selected substituted benzenes were computed and analyzed. After that, the equilibrium structures are described and three possible interaction motifs frequently found by experiment (namely through Bi⋅⋅⋅π arene, Cl⋅⋅⋅π arene or Bi⋅⋅⋅R interactions) are discussed. We also focus on the competition between dispersion and donor‐acceptor interactions in different structural arrangements. In addition to the investigation of structural parameters, nuclear magnetic shifts were computed to assess the usefulness of NMR spectroscopy to study bismuth trichloride interactions with aromatic compounds.

## Computational Details

All calculations were performed using a development version of the Orca 4.0.1 program.^32^, ^33^ Geometries were optimized using the PBE^34^, ^35^ density functional in combination with def2‐QZVP^36^ basis set and default effective core potentials (def2‐ECP) for bismuth replacing 60 electrons. Dispersion correction^37^ (D3) by Grimme with Becke‐Johnson (BJ) damping^38^ and fine integration grids (grid 4) were used in DFT calculations. Stationary points were confirmed by subsequent normal mode analysis and the resolution of the identity approximation was employed. The energies were further refined at the DLPNO‐CCSD(T)^22^, ^23^, ^24^, ^25^, ^26^, ^27^, ^28^ level of theory using the cc‐pVQZ^39^ basis set for lighter elements and cc‐pwCVQZ‐PP^40^ combined with the SK‐MCDHF‐RSC effective core potential for bismuth. TightPNO^41^, ^42^ settings were used in DLPNO‐CCSD(T) calculations. Local energy decomposition was performed on the minima on the potential energy surface and equilibrium structures in order to decompose the interaction energy obtained at the DLPNO‐CCSD(T) level of theory. The Foster‐Boys orbital localization method^43^ was used in coupled cluster calculations.

Local energy decomposition^29^, ^30^ (LED) is a method that is used to obtain accurate interaction energies between two or more fragments of the interacting system. The LED method uses the feature of the local correlation methods that localized occupied orbitals can be ascribed to the specific fragments of the molecule. The interaction energy obtained from the DLPNO‐CC calculations can be further decomposed into physically meaningful contributions arising from the correlation energy and the Hartree‐Fock reference. Generally, from the Hartree‐Fock interaction energy contributions such as electrostatic interaction, polarization and donor‐acceptor properties can be obtained. The interaction energy arising from the correlation energy corrects all the terms obtained at the HF level and additionally yields dispersive contributions. The computational cost of LED analysis within the DLPNO‐CC calculations is negligible.

In our previous work^20^ we benchmarked several dispersion‐corrected density functionals (B3LYP, PBE0, M062X, BP86, and PBE) as well as other methods like MP2 and DFT‐SAPT regarding their performance in studying pnictogen(III)⋅⋅⋅π arene interactions with reference to DLPNO‐CCSD(T) results. Especially PBE‐D3 yields results that are in a very good agreement with the DLPNO‐CCSD(T), therefore it was employed to optimize the geometries and perform potential energy surface scans.

NMR chemical shifts were computed using the M06L,^44^ TPSS,^45^ B3LYP,^46^, ^47^, ^48^ and KT2^49^ density functionals in conjunction with the pcSseg‐3^50^ basis set for lighter elements and the Sapporo‐DKH3‐TZP‐2012^51^ basis set for the bismuth atom.

## 2 Results and Discussion

### 2.1 Substituent Effects on Bi⋅⋅⋅π Arene Interaction in BiCl_3_ Adducts with Benzene Derivatives

#### 2.1.1 Idealized Structures

In order to assess the influence of the substitution atthe benzene ring on the interaction strength and the donor‐acceptor properties of the intermolecular interaction, the BiCl_3_⋅⋅⋅C_6_H_6_ adduct was chosen. This adduct exhibits the highest interaction energy and the most pronounced donor‐acceptor character (34 % of the total interaction energy) among all previously investigated systems.^20^

To study the influence of the aromatic substituent on interaction properties in BiCl_3_⋅⋅⋅C_6_H_6_ adducts, benzene derivatives with one or three electron donating or electron withdrawing substituents were chosen (Scheme 1). We assess if already one substituent can affect the interaction strength and if three substituents have further influence on the interaction properties. First, bismuth trichloride and substituted benzenes were optimized at the PBE‐D3/def2‐QZVP level of theory and idealized adducts were constructed to separate the pure Bi⋅⋅⋅π arene interaction from other possible interactions of the arene with bismuth trichloride. Idealized structures of van der Waals adducts were assembled and rigid distance scans were performed at the DFT‐D3 level of theory according to the procedure described previously.^20^ Initially, we selected a broader set of benzene derivatives with one or three substituents (R=CF_3_, NO_2_, NH_2_, OH, OCHO, F, Cl, CHO, CN, C_2_H_3_) in order to test various possible electronic effects of the substituents and performed DFT‐D3 rigid distance scans. As some of the substituents have the same effect on the interaction strength (see Figure S1 in the Supporting Information) we decided to narrow down the set of substituents and performed rigid distance scans at the DLPNO‐CCSD(T)/cc‐pVQZ (cc‐pwCVQZ‐PP for bismuth and TightPNO settings) level of theory for a limited number of benzene derivatives (R=CF_3_, NO_2_, NH_2_, OH, OCHO). Furthermore, local energy decomposition was performed to analyze the dispersive and non‐dispersive contributions to the interaction energy at the DLPNO‐CCSD(T) level of theory.

The Bi(CH_3_)_3_⋅⋅⋅benzene adduct exhibits a purely dispersive type of intermolecular interaction. Therefore, in order to analyze if substituents can influence the properties of the interaction in this type of adduct, substituents exhibiting strongest effects on the interaction potential were chosen. The rigid distance scans for Bi(CH_3_)_3_ adducts with nitrobenzene and aminobenzene were performed at the PBE‐D3/def2‐QZVP level of theory and the results were compared to the BiCl_3_⋅⋅⋅C_6_H_6_ adduct as reference (see Figure S2). Introduction of a nitro or amino group has almost no influence on the interaction strength, supporting the hypothesis that generally speaking the main influence is the π→σ* donor‐acceptor interaction which is absent in Bi(CH_3_)_3_ adducts. The results can be found in the Supporting Information.

**Figure 1:**
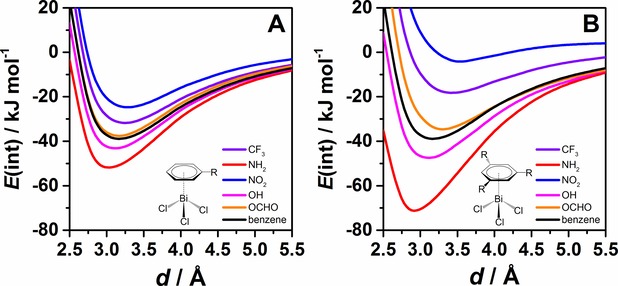
Potential energy curves (in kJ mol^−1^) for idealized BiCl_3_ adducts with benzene derivatives (see Scheme 1 for details) with A) one substituent and B) three substituents computed at the DLPNO‐CCSD(T)/cc‐pVQZ (cc‐pwCVQZ‐PP for bismuth) level of theory with tightPNO settings.

The interaction potentials for the BiCl_3_ adducts with selected substituted benzenes calculated at the DLPNO‐CCSD(T) level of theory are shown in Figure 1. Introducing one substituent in the benzene ring (see Figure 1A) already influences the interaction strength compared to the interaction potential of the BiCl_3_⋅⋅⋅C_6_H_6_ adduct. Substituents having the strongest influence on the interaction strength are amino and nitro groups. Introducing an amino group to the benzene ring increases the interaction energy from −40 kJ mol^−1^ (for benzene) to about −50 kJ mol^−1^. A nitro group decreases the interaction energy to about −25 kJ mol^−1^ compared to unsubstituted benzene. A hydroxyl group increases the interaction energy to about −45 kJ mol^−1^. The trifluoromethylbenzene adduct with BiCl_3_ exhibits an interaction strength that is decreased to about −30 kJ mol^−1^ compared to the BiCl_3_⋅⋅⋅C_6_H_6_ adduct. An ester group (OCHO) in the benzene ring only slightly decreases the interaction strength.

Introduction of three substituents in the benzene ring changes the interaction strength even further for most of the substituents studied here (see Figure 1B). Nitro and amino groups have the strongest effects. The presence of three amino groups increases the interaction energy to −70 kJ mol^−1^. Three nitro groups further reduce the interaction energy to about −5 kJ mol^−1^. Also exchanging hydrogen atoms with CF_3_ groups decreases the interaction strength quite significantly to −20 kJ mol^−1^. Introducing three hydroxy or three ester groups does not alter the interaction significantly compared to introducing one of these substituents (see Figure 1A and B).

**Figure 2:**
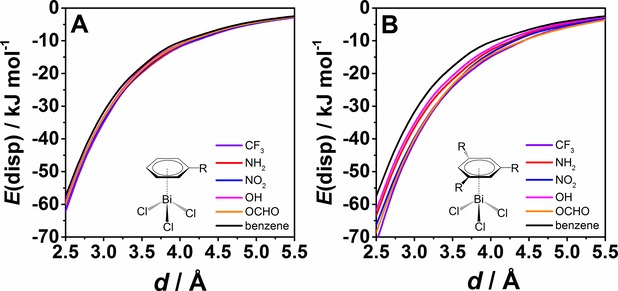
Dispersion energy contributions according to LED [DLPNO‐CCSD(T)/cc‐pVQZ (cc‐pwCVQZ‐PP for bismuth) with tightPNO settings] for the distance scans shown in Figure 1.

Figure 2 depicts the dispersion contribution of the interaction energies obtained from the local energy decomposition analysis at the DLPNO‐CCSD(T) level of theory. Figure 2A shows that the dispersion energy does not change much for benzenes with one substituent compared to unsubstituted benzene. By introducing three substituents in the benzene ring a change in dispersion energy becomes more noticeable (see Figure 2B) than in the case of one substituent. However, compared to the influence on the total interaction energies (±40 kJ mol^−1^) this is a small effect (+10 kJ mol^−1^ at most).

**Figure 3:**
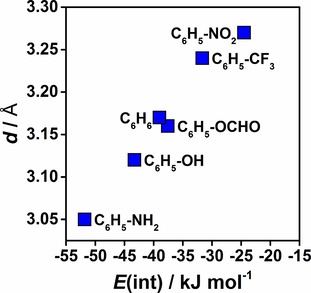
Correlation between interaction energy and the minimum distances on the potential energy surfaces for selected BiCl_3_⋅⋅⋅R−C_6_H_5_ adducts obtained at the DLPNO‐CCSD(T) level of theory.

Additionally, minima on the potential energy surfaces shown in Figure 1 were estimated by interpolation and analyzed by means of LED. Results are presented in Table 1. The equilibrium distances between the bismuth atom and the centroid of the arene ring differ from the distance estimated for the BiCl_3_⋅⋅⋅C_6_H_6_ adduct. Introducing electron donating substituents decreases the distance. This effect is strongest for the amino group (see Table 1). Electron withdrawing substituents increase the distance and this effect is most pronounced in the case of the nitrobenzene adduct. The correlation between minimum distances and interaction energies for adducts of monosubstituted benzene derivatives is shown in Figure 3. Again, these effects are more pronounced in the case of benzene derivatives with three substituents. Note that the introduction of electron withdrawing or electron donating substituents does not only affect the strength of the interaction but also its type. For instance, in the case of the NO_2_−C_6_H_5_ adduct the dispersion energy is the only attractive contribution to the total interaction energy (repulsive non‐dispersive interaction+attractive dispersion), while for the NH_2_−C_6_H_5_ adduct the dispersion energy amounts to 68 % of the total interaction energy. This effect is more pronounced for benzene derivatives with three substituents. Hence, mostly the non‐dispersive contribution is altered by substitution and its contribution increases for electron donating and decreases for electron withdrawing substituents. We will come back to the quantification and discussion of these effects in section 2.2.4.

**Table 1:** Interaction energies (in kJ mol^−1^) and their components of the minima on the potential energy surfaces obtained at the DLPNO‐CCSD(T) level of theory.

R	*d* [Å]	*E*(int)^[a]^≡*E*(tot)^[b]^	*E*(HF)^[c]^	*E*(disp)^[d]^	*E*(int‐disp)^[e]^
NO_2_	3.27	−24.5	11.6	−27.6	3.1
CF_3_	3.24	−31.7	3.8	−29.7	−2.0
OCHO	3.16	−37.6	−2.0	−30.7	−6.9
benzene	3.17	−39.0	−5.3	−25.7	−13.3
OH	3.12	−43.3	−5.3	−31.9	−11.4
NH_2_	3.05	−51.8	−9.1	−35.3	−16.5
3NO_2_	3.55	−3.9	33.3	−24.2	20.3
3CF_3_	3.42	−18.0	19.7	−28.6	10.6
3OCHO	3.30	−34.4	3.7	−33.2	−1.2
benzene	3.17	−39.0	−5.3	−25.7	−13.3
3OH	3.11	−47.6	−1.7	−35.0	−12.6
3NH_2_	2.94	−70.5	−14.0	−44.0	−26.6

[a] *E*(int): interaction energy. [b] *E*(tot): total electronic energy. [c] *E*(HF): interaction energy at the HF level of theory. [d] *E*(disp): dispersion energy. [e] *E*(int‐disp): interaction energy without dispersion contribution. Note that in these idealized structures the monomers are kept fixed upon dimer formation, hence the geometry preparation (deformation) energy *E*(geo‐prep) equals zero.

Charge transfer can be associated with non‐dispersive contributions (donor‐acceptor properties) to the interaction energies. Our previous studies show that charge is transferred from benzene to the MX_3_ molecule. Table S4 presents calculated partial natural charges for idealized minima on the potential energy surfaces. More charge is accumulated on BiCl_3_ when interacting with benzene derivatives with electron donating substituents [Σq(BiCl_3_)] compared with adducts of unsubstituted benzene. The charge is depleted on Bi and shifts towards chlorine atoms. This effect is stronger for systems with three substituents.

In summary, substituents in the benzene ring offer the possibility to enhance or decrease the BiX_3_⋅⋅⋅π arene interaction by a factor of two by modifying the donor‐acceptor interaction contribution. This is also confirmed by investigating the influence of the substituent on the interaction potential of Bi(CH_3_)_3_⋅⋅⋅benzene which does not act as a π acceptor and is independent of the substituent (see SI).

#### 2.1.2 Trends in the Periodic Table

In our previous work we examined trends for pnictogen⋅⋅⋅benzene adducts across the periodic table. It has been found that changing the pnictogen from arsenic to antimony has smaller influence on the interaction energies than exchanging antimony to bismuth. It was also shown that bismuth in BiX_3_ is more susceptible to the ligand effects of X than the other heavier pnictogens. This is reflected not only by the interaction strength but also by its character, i. e. increasing donor‐acceptor properties.

**Figure 4:**
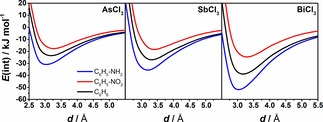
Comparison of interaction potentials for AsCl_3_, SbCl_3_ and BiCl_3_ adducts with substituted benzenes (R=NH_2_, NO_2_) computed at the DLPNO‐CCSD(T)/cc‐pVQZ (cc‐pwCVQZ‐PP for pnictogen; TightPNO) level of theory.

Figure 4 depicts interaction potentials for AsCl_3_, SbCl_3_ and BiCl_3_ adducts with nitro‐ and aminobenzene computed at the DLPNO‐CCSD(T) level of theory. Potential energy curves for the MCl_3_ interaction with benzene were also added for comparison. In the case of all pnictogen trichloride adducts addition of either the NH_2_ or the NO_2_ group changes the strength of the interaction. Alike the BiCl_3_ adduct, the interaction strength of AsCl_3_ and SbCl_3_ adducts with nitrobenzene is weaker than of their benzene adducts. In all cases an amino group increases the interaction strength. Among all studied systems, the AsCl_3_⋅⋅⋅nitrobenzene adduct is the most weakly interacting system and the change from arsenic to antimony is not as significant as going from antimony to bismuth, which correlates with the change in number of electrons. However, minimum distances on the AsCl_3_ potential energy curves are very similar to the minimum distances on the BiCl_3_ curves, while minimum distances on the SbCl_3_ curve are longer. Minima on the PES were also found by interpolation and LED analysis was performed (see Table 2). The minimum distance for aniline adducts with AsCl_3_ and BiCl_3_ is 3.05 Å, whereas the minimum distance for the SbCl_3_⋅⋅⋅aniline adduct is 3.18 Å (see Table 2 for details). The change in interaction energies from arsenic to antimony is small compared to the change from antimony to bismuth. The increase in interaction energies for pnictogen trichloride adducts with benzene derivatives goes along with the increasing van der Waals radius of a pnictogen but by analyzing the size of van der Waals radii the larger change would be expected when going from arsenic to antimony rather than from antimony to bismuth. Note that dispersion is the main contribution (see Table 2) to the interaction energy in the aminobenzene adducts of all pnictogen chlorides. In the case of nitrobenzene adducts, dispersion is the only attractive contribution to the interaction energy.

**Table 2:** Interaction energies (in kJ mol^−1^) and their components of the minima on the potential energy surfaces obtained at the DLPNO‐CCSD(T) level of theory.

Adduct	d [Å]	*E*(int)^[a]^≡*E*(tot)^[b]^	*E*(HF)^[c]^	*E*(disp)^[d]^	*E*(int‐disp)^[e]^
AsCl_3_⋅⋅⋅NO_2_C_6_H_5_	3.24	−17.8	14.0	−21.6	3.9
SbCl_3_⋅⋅⋅NO_2_C_6_H_5_	3.40	−18.4	14.4	−22.2	3.8
BiCl_3_⋅⋅⋅NO_2_C_6_H_5_	3.27	−24.5	11.6	−27.6	3.1
AsCl_3_⋅⋅⋅C_6_H_6_	3.17	−23.8	6.8	−21.7	−2.1
SbCl_3_⋅⋅⋅C_6_H_6_	3.31	−27.2	5.2	−22.4	−4.8
BiCl_3_⋅⋅⋅C_6_H_6_	3.17	−39.0	−5.3	−25.7	−13.3
AsCl_3_⋅⋅⋅NH_2_C_6_H_5_	3.05	−31.0	7.4	−27.8	−3.2
SbCl_3_⋅⋅⋅NH_2_C_6_H_5_	3.18	−35.7	5.0	−27.9	−7.8
BiCl_3_⋅⋅⋅NH_2_C_6_H_5_	3.05	−51.8	−9.1	−35.3	−16.5

[a] *E*(int): interaction energy. [b] *E*(tot): total electronic energy. [c] *E*(HF): interaction energy at the HF level of theory. [d] *E*(disp): dispersion energy. [e] *E*(int‐disp): interaction energy without dispersion contribution. Note that in these idealized structures the monomers are kept fixed upon dimer formation, hence the geometry preparation (deformation) energy *E*(geo‐prep) equals zero.

In the case of aminobenzene adducts of all pnictogens the non‐dispersive contributions are larger compared to benzene adducts. This is also reflected in charge shift from benzene derivatives to MCl_3_. Table S5 depicts the calculated natural partial charges for idealized structures of all pnictogen adducts studied here. The charge on MCl_3_ is decreased for nitrobenzene and increased for aniline adducts compared to the adducts of unsubstituted benzene.

### 2.2 Intermolecular Interactions of BiCl_3_: Comparison of Binding Motifs

The broad variety of experimentally characterized structures of the heavier pnictogen(III) compounds including π systems exhibits a wide range of interaction motifs. Besides donor‐acceptor interactions, M⋅⋅⋅π arene interactions are observed. However, also another motif of “tail‐to‐tail” interactions are present in crystal structures, but an understanding in which case a certain interaction motif will be predominant is still missing.

**Scheme 2:**
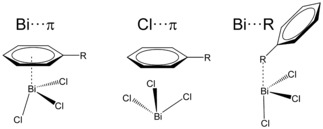
Three possible contact modes for BiCl_3_⋅⋅⋅benzene adducts.

Therefore, we studied three possible contact modes of R−C_6_H_5_⋅⋅⋅BiCl_3_ adducts as depicted in Scheme 2. In contrast to section 2.1, in which the focus was on the fundamental interaction mechanism for which idealized structures were used, here we focus on a variety of motifs where all geometries were fully optimized.

Structures of adducts denoted as Bi⋅⋅⋅π arene are based on the interaction mainly between the bismuth atom and benzene derivatives with small influence of chlorines while the structure of the Cl⋅⋅⋅π arene adduct consists only of interaction between benzene derivatives and chlorine atoms. Such purely dispersive interactions are often very important in the formation of molecular assemblies.^21^ We also assume that the interaction can occur between bismuth and the electron donating atom of the substituent on benzene. These adducts are denoted as Bi⋅⋅⋅R.

BiCl_3_ adducts with one substituent in the benzene ring were selected for further analysis. The structures of these adducts were optimized at the PBE‐D3/def2‐QZVP level of theory and local energy decomposition was performed at the DLPNO‐CCSD(T) level of theory. The structures of these adducts are shown and discussed in the following sections and the results of the LED analysis are shown in Tables 3, 4, and 5.

**Table 3:** Interaction energy components (in kJ mol^−1^) obtained at the DLPNO‐CCSD(T) level of theory for equilibrium structures of Bi⋅⋅⋅π arene adducts.

Adduct	Δ*E*(tot)	Δ*E*(geo‐prep)	Δ*E*(int)	Δ*E*(disp)	Δ*E*(int‐disp)
NO_2_	−26.7	2.0	−28.7	−28.1	−0.6
CN	−31.1	2.0	−33.1	−30.3	−2.8
CF_3_	−36.3	2.4	−38.7	−32.0	−6.7
CHO	−36.9	2.4	−39.3	−32.6	−6.7
F	−37.1	2.9	−40.0	−31.3	−8.7
Cl	−37.7	3.0	−40.8	−32.1	−8.6
OCHO	−39.4	4.7	−44.1	−34.1	−10.0
C_2_H_3_	−47.3	4.2	−51.5	−37.6	−13.9
OH	−49.2	7.7	−56.9	−38.8	−18.1
NH_2_	−61.9	7.4	−66.6	−42.5	−24.1
benzene	−42.3	3.7	−45.9	−23.7	−22.2

[a] Δ*E*(tot): total electronic energy. [b] Δ*E*(geo‐prep): geometrical preparation, deformation energy. [c] Δ*E*(int): interaction energy. [d] Δ*E*(disp): dispersion energy. [e] Δ*E*(int‐disp): interaction energy without dispersion contribution.

#### 2.2.1 Bi⋅⋅⋅π Arene Equilibrium Structures

**Figure 5:**
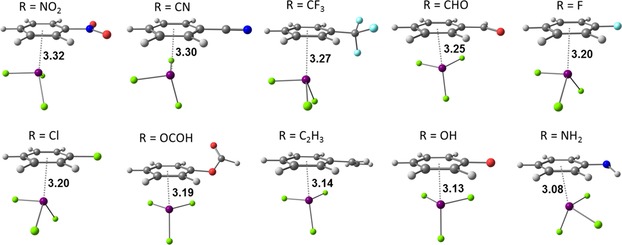
Equilibrium structures of Bi⋅⋅⋅π arene adducts optimized at the PBE‐D3/def2‐QZVP level of theory. Important intermolecular distances are given in Å.

As expected, the geometries of the equilibrium structures (see Figure 5) discussed in this part differ from the idealized structures discussed in section 2.1. The BiCl_3_ molecule is tilted with respect to plane of the arene ring and the substituents X interact with the π system as well. As reported by Hobza et al.,^52^ the structure of the SbCl_3_⋅⋅⋅toluene adduct, that is isostructural to the BiCl_3_⋅⋅⋅toluene adduct,^53^ is proposed to be a result of the attraction between the σ‐hole and the π system. In our previous work,^20^ where we studied intermolecular interactions of benzene with pnictogen compounds of the form MX_3_ (M=As, Sb, Bi and R=Cl, OCH_3_, CH_3_), we also found that the MX_3_ molecules are tilted. Our findings showed that this tilted structure is partially due to the interaction of X with the arene ring, especially important in the case of M(CH_3_)_3_ compounds. Analysis of the tilting potential energy curves and pair contributions of localized orbitals using the local energy decomposition revealed that mainly dispersion is responsible for the tilted geometries. Contrary to the results obtained by Hobza et al.,^52^ our analysis of equilibrium structures of the MCl_3_⋅⋅⋅benzene adducts showed that despite the more pronounced σ‐hole at the pnictogen, geometries of these adducts are less tilted towards the plane of benzene than their methyl and methoxy counterparts. This is due to the π→σ* charge transfer that results in the higher covalency of the bonding in the chloride adducts.

Interaction energies of the equilibrium structures of Bi⋅⋅⋅π arene adducts are a few kJ mol^−1^ larger than the interaction energies computed for the minima on the potential energy curves of idealized structures and range from −20 kJ mol^−1^ to −60 kJ mol^−1^. Only BiCl_3_ adducts with benzenes having C_2_H_3_, OH and NH_2_ substituents gave higher interaction energies than the adduct with unsubstituted benzene. Dispersion is still the largest contribution to the interaction energy for all adducts (see Table 3). Dispersion energy plots for the Bi⋅⋅⋅π arene adducts obtained from the LED analysis ca be found in the Supporting Information (see Figure S6). Values of geometry preparation energies are highest for the adducts with electron donating substituents and are highest for phenol and aniline (about 7.5 kJ mol^−1^).

**Figure 6:**
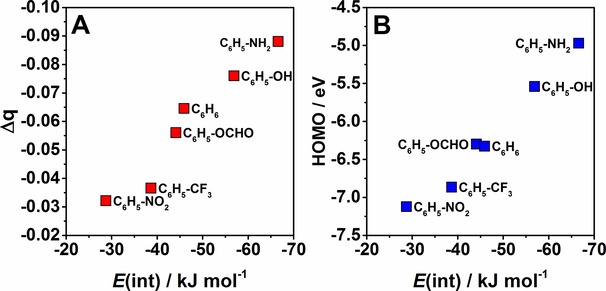
Correlation between the DLPNO‐CCSD(T) interaction energies and A) Δ*q* between bounded and unbounded BiCl_3_ molecule and B) HOMO energies obtained at the PBE‐D3 level of theory.

An analysis of calculated natural partial charges shows that the sum of the partial charges on the BiCl_3_ molecule changes upon interaction with benzene. The results are shown in Table S7 and Figure 6A. The charge concentration increases on the BiCl_3_ molecule with the increasing interaction energies of a given adduct. The highest charge is accumulated on the BiCl_3_ molecule when interacting with benzene derivatives carrying electron donating groups. An analysis of results in Table S7 shows that charge is shifted from bismuth to chlorine. This goes in line with analysis of molecular orbitals. When the HOMO (π orbital) of the benzene derivative is closer in energy to the LUMO (σ* orbital) of BiCl_3_ then more charge is transferred.

**Figure 7:**
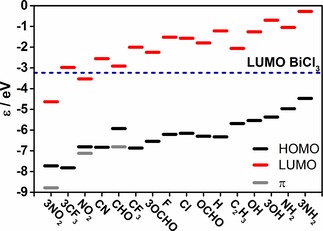
Energies of the frontier molecular orbitals (in eV) for studied benzenes and BiCl_3_ obtained at the PBE‐D3/def2‐QZVP level of theory. Note that the molecules on the horizontal axis are actually ordered by increasing Bi⋅⋅⋅π arene interaction strength from left to right. In the cases for which the HOMO orbital is not the π orbital, the energy of the highest π orbital is given as grey dash.

**Figure 8:**
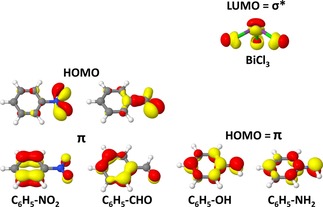
HOMO and π orbitals of selected substituted benzenes (R=NO_2_, CHO, OH, and NH_2_) and σ* of the BiCl_3_ molecule as obtained at the PBE‐D3/def2‐QZVP level of theory.

The energies of the frontier molecular orbitals calculated at the PBE‐D3/def2‐QZVP level of theory are summarized in Table S8 and Figure 7. Figure 7 depicts energies of HOMO, LUMO and selected π orbitals of substituted benzenes. For substituted benzene, the introduction of electron donating groups like NH_2_, OH and C_2_H_3_ shifts their HOMO orbital to higher energies while the presence of electron‐pulling groups like NO_2_ and CF_3_ shifts the HOMO/π orbitals to lower energies. Note that for most substituted benzenes the HOMO is a mixture of π and n orbitals. The energy gap between the HOMO of substituted benzene and the LUMO of BiCl_3_ becomes smaller which facilitates π→σ* charge transfer. Note that in the case of benzene derivatives with one or three NO_2_ and CHO the HOMO is actually a non‐bonding orbital (see Figure 8).

Figure 6 depicts the correlation between interaction energies and Δ*q* (Figure 6A) or HOMO or π orbital (Figure 6B) for substituted benzenes. Figure 6A shows that the interaction energies increase with the increasing charge concentration on BiCl_3_. The HOMO values shift to lower energies with the decreasing interaction energies for their adducts with BiCl_3_. Also the LUMO values shift to lower energies with the decreasing interaction energies (see Figure S9 in Supporting Information).

Hence, all analyses given above indicate that the strongest influence on the Bi⋅⋅⋅π arene interaction is the π→σ* donor‐acceptor interaction and its strength is determined by the relative energies of the donor and acceptor orbitals.

#### 2.2.2 Donor‐Acceptor Interaction with Functional Groups

**Figure 9:**
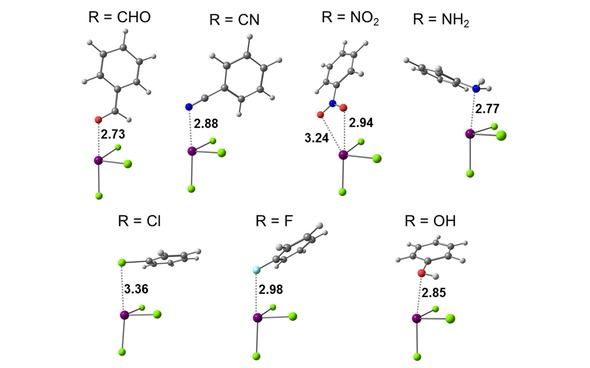
Optimized geometries (PBE‐D3/def2‐QZVP) of the Bi⋅⋅⋅R type intermolecular adducts of BiCl_3_ and benzene derivatives. Important intermolecular distances are given in Å.

In the previous section it was mentioned that for compounds with lone pair(s) on the substituent an orbital is accessible for donor‐acceptor interaction. In the cases where this orbital corresponds to the HOMO, coordination of the functional group to the bismuth atom is expected to be favorable. This implies a second type of interaction mode between the bismuth atom and a lone pair of electrons from the substituent itself. In this type of adducts no direct interaction with the π system is expected. Selected optimized Bi⋅⋅⋅R type intermolecular adducts are given in Figure 9.

The interaction energies of Bi⋅⋅⋅R adducts calculated at the DLPNO‐CCSD(T) level of theory vary from −28.5 kJ mol^−1^ for chlorobenzene to −66.4 kJ mol^−1^ for aniline (see Table 4). The distances between the bismuth atom and the donor atom of the substituent are shorter than 3 Å, except for the adduct with chlorobenzene (*r*=3.36 Å). Thus, the intermolecular distances between the bismuth atom and the lone pair of electrons is much shorter (by more than 1 Å) than the sum of van der Waals radii (>4 Å) of bismuth and the particular donor atom (O, N, F, and Cl) (see Table S10).

**Table 4:** Interaction energy components (in kJ mol^−1^) obtained at the DLPNO‐CCSD(T) level of theory for equilibrium structures of Bi⋅⋅⋅R adducts.

Adduct	Δ*E*(tot)	Δ*E*(geo‐prep)	Δ*E*(int)	Δ*E*(disp)	Δ*E*(int‐disp)
NO_2_	−41.1	−5.0	−46.1	−24.5	−21.6
CN	−44.7	−4.5	−49.2	−27.2	−22.0
CHO	−51.1	−6.9	−58.0	−25.7	−32.3
F	−25.6	−2.9	−28.6	−19.8	−8.8
Cl	−26.4	−2.2	−28.5	−25.9	−2.7
OH	−40.7	−5.4	−46.1	−27.8	−18.4
NH_2_	−58.8	−7.6	−66.4	−32.5	−33.9

[a] Δ*E*(tot): total electronic energy. [b] Δ*E*(geo‐prep): geometrical preparation, deformation energy. [c] Δ*E*(int): interaction energy. [d] Δ*E*(disp): dispersion energy. [e] Δ*E*(int‐disp): interaction energy without dispersion contribution.

Comparing the interaction energies with those of the Bi⋅⋅⋅π arene motif reveals that there are actually several cases in which the Bi⋅⋅⋅R motif is the more stable one, which are the adducts with nitrobenzene, benzaldehyde and benzonitrile. A glance at Figure 7 shows that these are actually the cases in which the corresponding highest donor orbital is not the π, but a non‐bonding orbital. Furthermore, the LED of the obtained structures indicates that the typical size of interaction energies for this bonding motif is smaller than for the Bi⋅⋅⋅π arene motif due to a reduced dispersion contribution, as can be expected from the reduced intermolecular contact area in these structures.

#### 2.2.3 Cl⋅⋅⋅π Arene Equilibrium Structures

**Figure 10:**
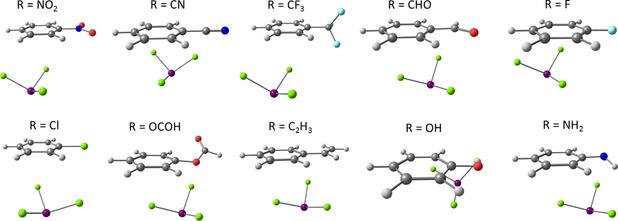
Equilibrium structures of Cl⋅⋅⋅π arene adducts optimized at the PBE‐D3/def2‐QZVP level of theory.

Another interaction motif would be to disregard the donor‐acceptor interaction and maximize the dispersion interaction. As halides can serve as good dispersion energy donors we also studied BiCl_3_⋅⋅⋅benzene adducts with interaction between the chlorine atoms and the π arene as investigated here, which could be termed “tail‐to‐tail” configuration. The relaxed structures of Cl⋅⋅⋅π arene adducts are shown in Figure 10 and the interaction energies and their components are shown in Table 5. Note that for each studied system, a Cl⋅⋅⋅π arene adduct was identified as a local minimum. Indeed, the character of the interaction in Cl⋅⋅⋅π arene adducts is purely dispersive. However, the interaction energies of these adducts are in the range of only from −15 kJ mol^−1^ to −23 kJ mol^−1^ and the dispersion contributions to the interaction energies are very similar for all of these adducts and are in the range from −20 kJ mol^−1^ to −26 kJ mol^−1^. Furthermore, the geometry preparation values are very small and are in the range from 0 kJ mol^−1^ to 2 kJ mol^−1^. The dispersion energy plots for Cl⋅⋅⋅π arene adducts obtained from LED analysis can be found in the Supporting Information (see Figure S12).

**Table 5:** Interaction energy components (in kJ mol^−1^) obtained at the DLPNO‐CCSD(T) level of theory for equilibrium structures of Cl⋅⋅⋅π arene adducts

Adduct	Δ*E*(tot)	Δ*E*(geo‐prep)	Δ*E*(int)	Δ*E*(disp)	Δ*E*(int‐disp)
NO_2_	−18.09	0.77	−18.86	−22.48	3.62
CN	−18.10	0.34	−18.04	−23.51	5.46
CF_3_	−16.65	1.69	−18.33	−22.60	4.27
CHO	−16.23	0.26	−16.50	−23.37	6.87
F	−14.48	0.73	−15.21	−21.36	6.15
Cl	−15.16	0.35	−15.51	−22.85	7.33
OCHO	−15.47	2.02	−17.50	−24.21	6.71
C_2_H_3_	−15.00	0.65	−15.64	−25.40	9.76
OH	−19.01	1.48	−20.49	−20.71	0.23
NH_2_	−19.80	0.38	−20.19	−26.19	6.00

[a] Δ*E*(tot): total electronic energy. [b] Δ*E*(geo‐prep): geometrical preparation, deformation energy. [c] Δ*E*(int): interaction energy. [d] Δ*E*(disp): dispersion energy. [e] Δ*E*(int‐disp): interaction energy without dispersion contribution.

In comparison to the other bonding motifs it can hence be concluded that the purely dispersive Cl⋅⋅⋅π arene interaction is not likely to compete with the other motifs due to its low interaction energies caused by the absence of the donor‐acceptor component.

#### 2.2.4 Balance between Donor‐Acceptor and Dispersion Interaction

After having analyzed the different interaction motifs discussed in the previous sections, we now address the question, how dispersion and donor‐acceptor interaction balance in different cases and what determines which bonding motif is predominant.

**Figure 11:**
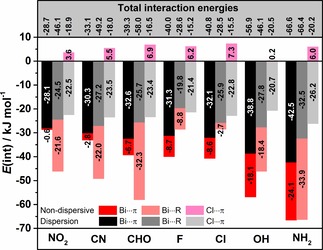
Comparison of the interaction energies and their dispersive and non‐dispersive contributions for selected Bi⋅⋅⋅π arene, Bi⋅⋅⋅R, and Cl⋅⋅⋅π arene type intermolecular adducts obtained at the DLPNO‐CCSD(T)/cc‐pVQZ (cc‐pwCVQZ‐PP for bismuth; TightPNO) level of theory.

The dispersive and non‐dispersive energy contributions to the interaction energies for Bi⋅⋅⋅π arene, Cl⋅⋅⋅π arene and Bi⋅⋅⋅R adducts are shown in Figure 11. Generally, the dispersion contribution to the interaction energies is higher in the case of Bi⋅⋅⋅π arene adducts. In the case of most Bi⋅⋅⋅R adducts this is compensated by a higher contribution of non‐dispersive i. e. donor‐acceptor interactions to the overall interaction energy. Exceptions are fluorobenzene and phenol adducts that have equally high donor‐acceptor interactions and also the adduct of chlorobenzene with an even smaller non‐dispersive energy contribution than in its Bi⋅⋅⋅π arene counterpart. Aniline adducts have identical interaction energies, but the composition of the interaction is different. The Bi⋅⋅⋅π arene complex of aniline has 10 kJ mol^−1^ more dispersive while its Bi⋅⋅⋅R adduct has more non‐dispersive contributions. Bi⋅⋅⋅R type adducts compete with Bi⋅⋅⋅π arene adducts and sometimes they even prevail. The situation is different for Cl⋅⋅⋅π arene adducts. The only attractive contribution to the interaction energy is dispersion. The dispersion energies of the Cl⋅⋅⋅π arene adducts are similar for all substituents and are lower compared to the Bi⋅⋅⋅π arene and Bi⋅⋅⋅R adducts.

**Figure 12:**
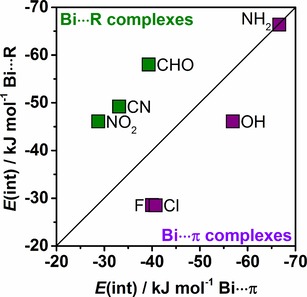
Correlation between DLPNO‐CCSD(T) interaction energies computed for Bi⋅⋅⋅π arene and Bi⋅⋅⋅R intermolecular adducts.

Figure 12 depicts the correlation between interaction energies of Bi⋅⋅⋅π arene, and Bi⋅⋅⋅R adducts. The graph classifies which type of adduct is more stable. Adducts with substituents like F, Cl, and OH are more stable as Bi⋅⋅⋅π arene while adducts of benzene derivatives with NO_2_, CN or CHO as substituent tend to form Bi⋅⋅⋅R adducts. Aniline is somewhere in between. Both its Bi⋅⋅⋅π arene and Bi⋅⋅⋅R adducts are isoenergetic.

Comparing systems in which the Bi⋅⋅⋅R motif is the most stable with systems for which the Bi⋅⋅⋅π arene motif is more stable a clear trend can be observed. In the first case, the donor‐acceptor contribution is comparably large due to the interaction with a more potent donor orbital. At the same time, the exclusive interaction with the substituent alone decreases the dispersion contribution. So in this case, the localization of the donor orbital on the substituent determines the interaction motif, but overall the Bi⋅⋅⋅R interaction strength is smaller, as it opposes a structural alignment optimal for dispersion. For the other systems, the donor orbital is the delocalized π system, so that in addition to a donor‐acceptor interaction the dispersion adds to the overall interaction, yielding larger overall interaction energies.

### 2.3 Spectroscopic Probes for Intermolecular Interactions

Nuclear magnetic resonance is a powerful tool that can be used to investigate intermolecular interactions due to its sensitivity. In order to obtain a more detailed experimental characterization of Bi⋅⋅⋅π arene interactions it would be desirable to have a spectroscopic probe for these systems, like for example the change of the ^13^C nuclear magnetic resonance upon coordination of an acceptor molecule. In order to assess whether the NMR chemical shifts could be used as indicator for Bi⋅⋅⋅π arene coordination, we performed calculations of the NMR chemical shifts for a series of adducts.

NMR chemical shifts of relaxed BiCl_3_ adducts with benzene derivatives having one substituent were calculated using the M06 L, TPSS, B3LYP and KT2 density functionals in conjunction with the pcSseg‐3 basis set in order to check if spectroscopic experiments can be performed and yield information on the intermolecular interaction. Chemical shifts were computed for Bi⋅⋅⋅π arene, Bi⋅⋅⋅R and Cl⋅⋅⋅π arene type adducts to analyze the influence of the conformation (due to possible dynamic effects in the solvent) and interaction type on the δ ^13^C values. Table 6 presents Δδ values that are the differences between computed isotropic chemical shifts of adducts and free substituted benzenes. Results shown in Table 6 were obtained using the M06 L functional as using other afore‐mentioned functionals yielded very similar results. Results obtained for other functionals can be found in Table S14 in the Supporting Information. Generally, in the case of Bi⋅⋅⋅π arene and Bi⋅⋅⋅R adducts the larger Δδ are observed [up to 10 ppm for Bi⋅⋅⋅π arene and 6 ppm (ring‐carbon) and 18 ppm (CN group) for Bi⋅⋅⋅R] than in the case of Cl⋅⋅⋅π arene adducts (maximum Δδ is almost 3 ppm).

**Table 6:** Computed gas phase Δδ values (in ppm) for substituted benzenes at the M06 L/pcSseg‐3 level of theory.

	Δδ *ipso*	Δδ *ortho*	Δδ *meta*	Δδ *para*	Δδ X1	Δδ X2
Bi⋅⋅⋅π arene						
C_2_H_3_	−6.20	−2.87	−3.69	−2.55	3.06	−10.08
CF_3_	−4.65	−3.70	−1.40	−2.29		
CHO	−1.86	−1.65	−3.18	−1.67	1.34	
Cl	−7.71	−4.83	−2.51	0.76		
CN	−5.24	−4.00	−1.41	−1.38	1.27	
F	−2.99	−5.27	−2.03	1.44		
NH_2_	3.22	−5.97	−5.93	−6.55		
NO_2_	−3.86	−3.98	−0.61	−0.77		
OCHO	−9.97	−2.54	−3.45	−2.73		
OH	−3.10	−4.26	−4.42	−4.62

Cl⋅⋅⋅π arene						
C_2_H_3_	−0.19	0.02	−0.13	−069	0.10	−0.95
CF_3_	0.61	−0.67	−0.24	−0.51		
CHO	−0.24	−0.51	−0.27	−0.07	−1.04	
Cl	−0.19	−0.25	−1.04	0.37		
CN	−0.36	−0.56	−0.89	0.20	−0.03	
F	−0.01	−0.98	−0.92	−0.62		
NH_2_	−1.04	−0.62	0.15	−1.01		
NO_2_	0.01	−1.00	−0.72	−0.41		
OCHO	−0.44	1.01	0.28	0.52		
OH	−0.70	−1.66	0.22	0.05

Bi⋅⋅⋅R						
CHO	2.65	−2.43	−0.40	−2.03	−5.37	
Cl	5.53	−1.39	−0.34	−2.59		
CN	5.48	0.49	−0.10	−2.01	−18.38	
F	0.76	−0.26	−0.06	−2.40		
NH_2_	4.56	−3.14	−1.31	−6.79		
NO_2_	6.03	0.30	−0.79	−0.62		
OH	5.17	−1.54	−0.34	−3.92

**Figure 13:**
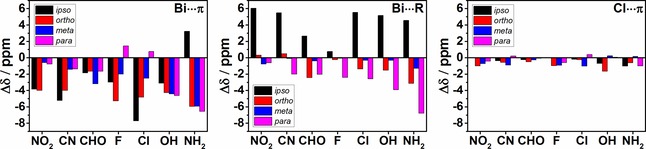
Comparison of the Δδ values (in ppm) of ^13^C calculated at the M06L/pcSseg‐3 level of theory.

Figure 13 depicts changes in calculated ^13^C NMR chemical shifts for all interaction modes studied here. The values of ^13^C Δδ for *ortho* and *meta* positions are averaged. There are no pronounced trends in the Δδ values. However, upon Bi⋅⋅⋅π interaction almost all ^13^C NMR signals shift upfield. In the case of Bi⋅⋅⋅R adducts chemical shifts of *ipso* carbon atoms are shifted downfield and almost all other ^13^C NMR signals are shifted to high field. For Cl⋅⋅⋅π arene adducts almost all signals are shifted to high field but the changes in Δδ values are very small compared to the Δδ values of Bi⋅⋅⋅π arene and Bi⋅⋅⋅R adducts.

From these numbers it seems possible that information about the coordination and the donor‐acceptor interaction could be obtained by NMR spectroscopy. However, as we have discussed above, other interaction motifs will compete with the Bi⋅⋅⋅π arene coordination. Preliminary experimental studies indicate that no pronounced changes and no obvious trends of specific signal shifts are observed, most likely as a result of averaged signals due to fast exchange between different structures and exchange with solvent (see Tables S15–S17 in the Supporting Information and Table S18 for a study using implicit an estimate for explicit solvent effects). Note that suitable solvents for this type of compounds contain potent donor groups and as a consequence they form intermolecular adducts which compete with adducts of benzene derivatives. Figure S20 depicts possible interaction modes between the BiCl_3_ molecule and nitromethane used as a solvent in the above‐mentioned NMR experiments. The interaction energy for nitromethane⋅⋅⋅BiCl_3_ adducts are larger than −40 kJ mol^−1^. Hence, the interaction energies between BiCl_3_ and the solvent molecule are in the same range as the interaction energies of most of the adducts of benzene derivatives. As a consequence, competition between solvent molecules, which are present in large excess, and the benzene derivative in forming intermolecular adducts is very likely to occur. For that reason, other techniques like solid state NMR or DOSY experiments as successfully applied for example to frustrated Lewis pair interactions^54^ might be more promising.

## 3 Conclusions

In this work we studied the interactions between pnictogen(III) compounds and substituted benzenes by means of computational chemistry methods like DFT‐D3, DLPNO‐CCSD(T) and local energy decomposition (LED). For this purpose BiCl_3_ and benzene derivatives with one or three substituents R (R=CF_3_, NO_2_, NH_2_, OH, OCHO) were chosen as model systems.

The interaction strength of BiCl_3_⋅⋅⋅π arene adducts can be modified by introduction of substituent(s) in the benzene ring. By introducing one substituent, the interaction energy, which is in the range of a few up to −60 kJ mol^−1^, can be altered by more than 30 %. Substitution actually alters the donor‐acceptor contribution to the interaction, while the dispersion interaction energy is less susceptible to structural changes. For instance, a nitro group decreases the interaction strength and changes the character of the interaction from a mixture of dispersion and donor‐acceptor to purely dispersive. On the other hand, the interaction energy of the BiCl_3_⋅⋅⋅aniline adduct is much higher than the interaction energy of the BiCl_3_⋅⋅⋅benzene adduct as introduction of an amino group enhances the donor‐acceptor interaction. Still, dispersion is the main component of the interaction for all studied adducts and it covers at least 60 % of the interaction energy. Accordingly, the purely dispersive pnictogen⋅⋅⋅π adducts (like the Bi(CH_3_)_3_⋅⋅⋅benzene adduct) are insensitive to introduction of substituents in the benzene ring.

Alteration of the donor‐acceptor properties occurs mainly due to the π→σ* charge transfer from the lowest π orbital of the aromatic compound to the empty σ* orbital of BiCl_3_. This effect is stronger for adducts of benzene derivatives with electron donating substituents which tend to shift the π donor orbital to higher energies and in consequence minimizing the energy gap between contributing orbitals and *vice versa*. This is also reflected in the charge transfer which correlates with the interaction energies – the larger the charge transfer, the higher the interaction energy.

Also in the case of AsCl_3_ and SbCl_3_ M⋅⋅⋅π arene adducts introduction of only one substituent (NH_2_ or NO_2_) already alters the interaction strength and properties. However, the change is small when going from arsenic to antimony and larger when going from antimony to bismuth.

Additionally to the Bi⋅⋅⋅π arene interaction motif, two other possible contact modes were studied for BiCl_3_⋅⋅⋅benzene adducts. The first one, the Cl⋅⋅⋅π arene, is the interaction between the π system and the substituents on bismuth. The Cl⋅⋅⋅π arene adducts have lower interaction energies compared to Bi⋅⋅⋅π arene adducts and the character of the interaction is purely dispersive.

The second one, Bi⋅⋅⋅R, is based on the interaction between bismuth and a donor atom of the substituent. The interaction in Bi⋅⋅⋅R adducts, just like in Bi⋅⋅⋅π arene adducts, is a balance of dispersion and donor‐acceptor component. Most of the Bi⋅⋅⋅R adducts have a higher non‐dispersive contribution than Bi⋅⋅⋅π arene adducts. Detailed analysis of molecular orbitals revealed that if the HOMO of a benzene derivative is a lone pair non‐bonding orbital of the donor atom in the substituent (O, N, Cl, F) it is likely that the Bi⋅⋅⋅R motif will compete with the Bi⋅⋅⋅π arene motif and in particular instances (CN, NO_2_, CHO) prevails.

Calculations of ^13^C NMR chemical shifts were performed on the equilibrium structures for all three binding motifs. The greatest influence on the ^13^C chemical shifts are found for the Bi⋅⋅⋅π arene and Bi⋅⋅⋅R adducts.

Generally, the choice of a substituent at the benzene derivative ring has a significant influence on the interaction strength and structure of the adducts formed with BiCl_3_. This trend is stronger than for the variation of substituents on the pnictogen atom.

Our results presented here demonstrate that by the interplay between dispersion energy contribution and donor‐acceptor properties the interaction strength within adducts of heavier pnictogens with benzene derivatives can be altered. This might hold implications for biological systems and catalysis, and allows to make use of this weak interaction to build up supramolecular architectures.

## Conflict of interest

The authors declare no conflict of interest.

## Supporting information

As a service to our authors and readers, this journal provides supporting information supplied by the authors. Such materials are peer reviewed and may be re‐organized for online delivery, but are not copy‐edited or typeset. Technical support issues arising from supporting information (other than missing files) should be addressed to the authors.

Supplementary
