# Supplementary material for: Balancing Donor‐Acceptor and Dispersion Effects in Heavy Main Group Element π Interactions: Effect of Substituents on the Pnictogen⋅⋅⋅π Arene Interaction
Source: Chemphyschem. 2019 Sep 12;20(19):2539–52. doi: 10.1002/cphc.201900747 (PMC6790748; doi:10.1002/cphc.201900747)
Supplement: Supplementary file 1 — Supplementary [file CPHC-20-2539-s001.pdf]

CHEMPHYSCHEM

Supporting Information

© Copyright Wiley-VCH Verlag GmbH & Co. KGaA, 69451 Weinheim, 2019

Balancing Donor-Acceptor and Dispersion Effects in Heavy Main Group Element π Interactions: Effect of Substituents on the Pnictogen···π Arene Interaction

Małgorzata Krasowska, Ana-Maria Fritzsche, Michael Mehring, and Alexander A. Auer*©2019 The Authors. Published by Wiley-VCH Verlag GmbH & Co. KGaA.
This is an open access article under the terms of the Creative Commons Attribution License, which permits use, distribution and reproduction in any medium, provided the original work is properly cited.

Table of Contents

Figure S1. Potential energy curves (in kJ mol^{-1}) for idealized $BiCl_3$ adducts with benzene derivatives (see Scheme 1 for details) a) with one substituent, b) with three substituents calculated at the PBE-D3/def2-QZVP level of theory. 4

Figure S2. Potential energy curves (in kJ mol^{-1}) for the interaction potentials of $Bi(CH_3)_3$ adduct with selected benzene derivatives calculated at the PBE-D3/def2-QZVP level of theory. 4

Table S3. Calculated polarizabilities (a.u.) of selected substituted benzenes at the PBE-D3/def2-QZVP level of theory. 5

Table S4. NBO partial charges (a.u.) for idealized $BiCl_3$ adducts with substituted benzenes computed at the PBE-D3/def2-QZVP level of theory. 5

Table S5. NBO partial charges (a.u.) for idealized MCl_3 adducts with selected substituted benzenes computed at the PBE-D3/def2-QZVP level of theory.. 6

Figure S6. Dispersion energy plots for equilibrium structures of Bi···π arene adducts computed at the DLPNO-CCSD(T)/cc-pVQZ (cc-pwCVQZ-PP for bismuth) level of theory with tightPNO settings. 7

Table S7. NBO partial charges (a.u.) for relaxed Bi···π arene adducts computed at the PBE-D3/def2-QZVP level of theory. 8

Table S8. Energies of the frontier molecular orbitals (eV) computed at the PBE-D3/def2-QZVP level of theory. 8

Figure S9. Correlation between energies of the LUMO and the DLPNO-CCSD(T) interaction energies of the minima on the potential energy curves. 9

Table S10. Sum of experimentally determined van der Waals radii (in Å) of bismuth and donor atoms (O, N, F, and Cl) of substituents. 9

Table S11. NBO partial charges (a.u.) for relaxed Bi···R adducts computed at the PBE-D3/def2-QZVP level of theory. 9

Figure S12. Dispersion energy plots for equilibrium structures of Cl···π arene adducts computed at the DLPNO-CCSD(T)/cc-pVQZ (cc-pwCVQZ-PP for bismuth) level of theory with tightPNO settings. 10

Scheme S13. Numbering of the atoms in NMR calculations.. 10

Table S14. Δδ (ppm) values (gas phase) for selected benzene derivatives with one substituent computed using various density functionals with the pcsSeg-3 basis set. 11

Table S15. ^{13}C NMR chemical shifts of the free monosubstituted arenes (C_6H_5R) and mixtures with $BiCl_3$ in a 1:1 and 1:7 molar ratio, measured in CD_3NO_2 solution at ambient temperature. 12

Table S16. The difference between the chemical shift of the arene in the mixture with $BiCl_3$ and the chemical shift of the free monosubstituted arene. 13

Table S17. ^{13}C NMR chemical shifts of the free trisubstituted arenes ($C_6H_3R_3$-1,3,5) and mixtures with $BiCl_3$ and in a 1:1 and 1:7 molar ratio, measured in CD_3NO_2 solution at ambient temperature. The difference between the chemical shift of the $BiCl_3$ adduct and the chemical shift of the free trisubstituted arene. 14

Table S18. Δδ values for nitrobenzene computed at the M06L/pcSseg-3 level of theory in the gas phase, with CPCM solvation model and with explicit solvent molecules. 15

Figure S19. Nitrobenzene molecule surrounded by explicit nitromethane molecules optimized at the PBE-D3/def2-SVP level of theory. 15

Figure S20. Possible motifs of interaction between solvent and $BiCl_3$ molecule as calculated at the PBE-D3/def2-QZVP level of theory. 15

Experimental details of NMR measurements........ 16

Cartesian coordinates 17

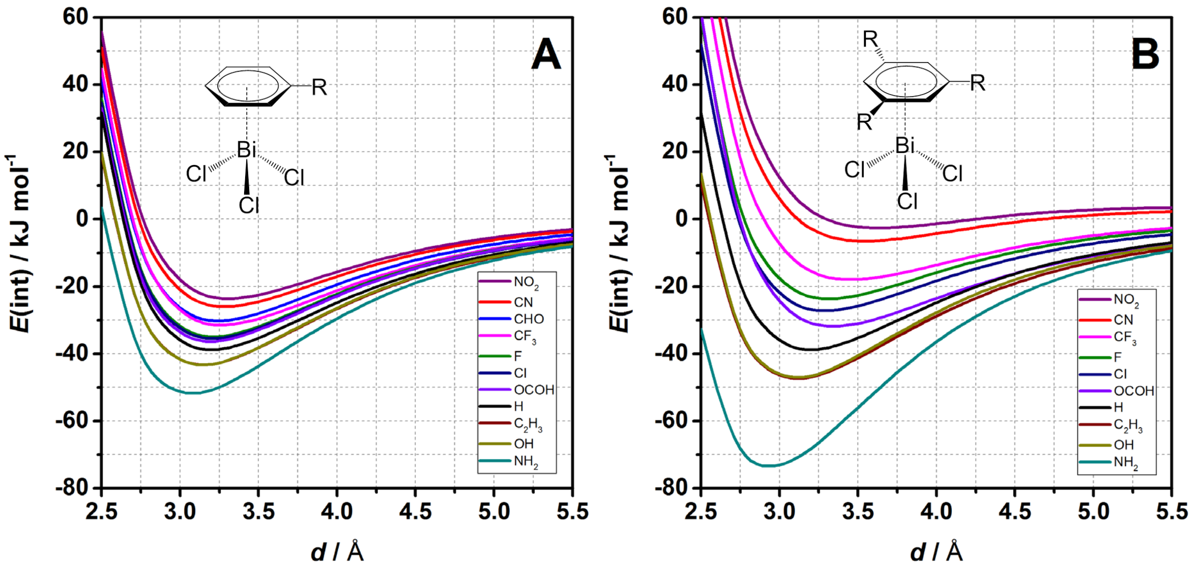

Figure S1. Potential energy curves (in kJ mol^{-1}) for idealized $BiCl_3$ adducts with benzene derivatives (see Scheme 1 for details) a) with one substituent, b) with three substituents calculated at the PBE-D3/def2-QZVP level of theory.

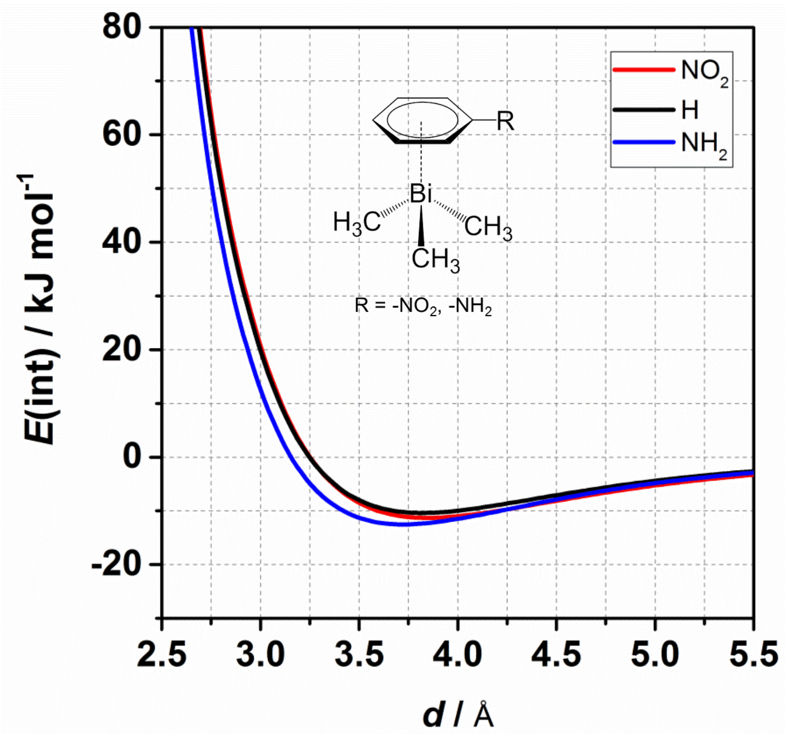

Figure S2. Potential energy curves (in kJ mol^{-1}) for the interaction potentials of $Bi(CH_3)_3$ adduct with selected benzene derivatives calculated at the PBE-D3/def2-QZVP level of theory.

Table S3. Calculated polarizabilities (a.u.) of selected substituted benzenes at the PBE-D3/def2-QZVP level of theory.

Molecule	Polarizability (a.u.)
CF_3	84.3
OCHO	90.3
NO_2	88.7
OH	75.4
CHO	88.1
F	69.6
NH_2	82.0
benzene	69.0

Table S4. NBO partial charges (a.u.) for idealized $BiCl_3$ adducts with substituted benzenes computed at the PBE-D3/def2-QZVP level of theory.

$BiCl_3$ adduct with	$\Sigma q(BiCl_3)$(adduct)	q(Bi)(adduct)	Δq(Bi)	$\Delta q(Cl_3)$
NO_2	-0.039	1.273	-0.001	-0.039
CF_3	-0.045	1.286	0.012	-0.057
OCHO	-0.059	1.287	0.013	-0.072
benzene	-0.059	1.289	0.015	-0.074
OH	-0.072	1.290	0.016	-0.088
NH_2	-0.089	1.292	0.018	-0.107
$3NO_2$	-0.009	1.231	-0.043	0.034
$3CF_3$	-0.019	1.263	-0.011	-0.009
3OCHO	-0.041	1.279	0.005	-0.046
3OH	-0.080	1.283	0.009	-0.089
$3NH_2$	-0.132	1.284	0.011	-0.143

Δq=q(adduct)-q(free). q – partial charge on a specific atom(s), Δq – difference between partial charge of an atom in $BiCl_3$ adduct and in an unbound $BiCl_3$ molecule.

Table S5. NBO partial charges (a.u.) for idealized MCl_3 adducts with selected substituted benzenes computed at the PBE-D3/def2-QZVP level of theory.

Adduct	$\Sigma q(MCl_3)$(adduct)	q(M)(adduct)	$\Delta q(M)$	$\Delta q(Cl_3)$
$AsCl_3\cdots NO_2C_6H_5$	-0.020	0.862	0.007	-0.027
$SbCl_3\cdots NO_2C_6H_5$	-0.023	1.177	-0.002	-0.021
$BiCl_3\cdots NO_2C_6H_5$	-0.039	1.273	-0.001	-0.038
$AsCl_3\cdots C_6H_6$	-0.032	0.877	0.022	-0.054
$SbCl_3\cdots C_6H_6$	-0.038	1.195	0.016	-0.055
$BiCl_3\cdots C_6H_6$	-0.059	1.289	0.015	-0.074
$AsCl_3\cdots NH_2C_6H_5$	-0.055	0.888	0.033	-0.088
$SbCl_3\cdots NH_2C_6H_5$	-0.063	1.196	0.018	-0.081
$BiCl_3\cdots NH_2C_6H_5$	-0.089	1.292	0.018	-0.107

Δq=q(adduct)-q(free)

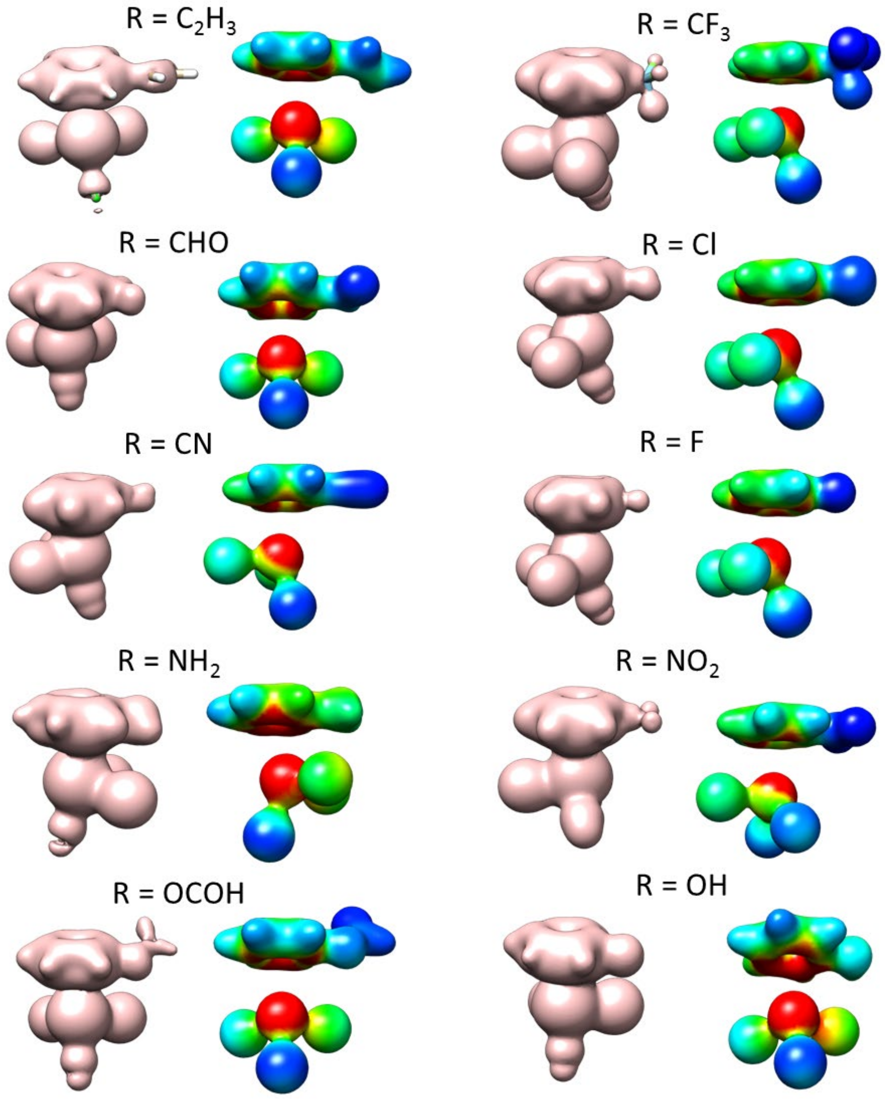

Figure S6. Dispersion energy plots for equilibrium structures of Bi···π arene adducts computed at the DLPNO-CCSD(T)/cc-pVQZ (cc-pwCVQZ-PP for bismuth) level of theory with tightPNO settings.

Table S7. NBO partial charges (a.u.) for relaxed Bi···π arene adducts computed at the PBE-D3/def2-QZVP level of theory.

$BiCl_3$ adduct with	$\Sigma q(BiCl_3)$(adduct)	q(Bi) (adduct)	Δq(Bi)	$\Delta q(Cl_3)$
NO_2	-0.032	1.283	0.009	-0.041
CN	-0.032	1.287	0.013	-0.045
CF_3	-0.037	1.296	0.022	-0.059
CHO	-0.039	1.292	0.019	-0.057
F	-0.058	1.287	0.013	-0.071
Cl	-0.050	1.292	0.018	-0.068
OCHO	-0.056	1.290	0.016	-0.072
C_2H_3	-0.061	1.301	0.028	-0.089
OH	-0.076	1.297	0.023	-0.099
NH_2	-0.088	1.303	0.029	-0.117

Δq=q(adduct)-q(free)

Table S8. Energies of the frontier molecular orbitals (eV) computed at the PBE-D3/def2-QZVP level of theory.

Molecule	HOMO/π orbital / eV	LUMO / eV	Δε (π→σ*)
$3NO_2$	-7.73 / -8.78	-4.64	-5.55
$3CF_3$	-7.82	-2.98	-4.58
NO_2	-6.81 / -7.12	-3.53	-3.89
CN	-6.83	-2.56	-3.59
CF_3	-6.87	-2.00	-3.63
CHO	-5.93 / -6.81	-2.92	-3.57
3OCHO	-6.54	-2.25	-3.30
F	-6.21	-1.52	-2.97
Cl	-6.15	-1.57	-2.92
OCHO	-6.30	-1.80	-3.06
C_6H_6	-6.33	-1.22	-3.53
C_2H_3	-5.69	-2.06	-2.45
OH	-5.54	-1.26	-2.30
3OH	-5.37	-0.70	-2.13
NH_2	-4.97	-1.05	-1.73
$3NH_2$	-4.47	-0.28	-1.23
$BiCl_3$	-7.66	-3.24	

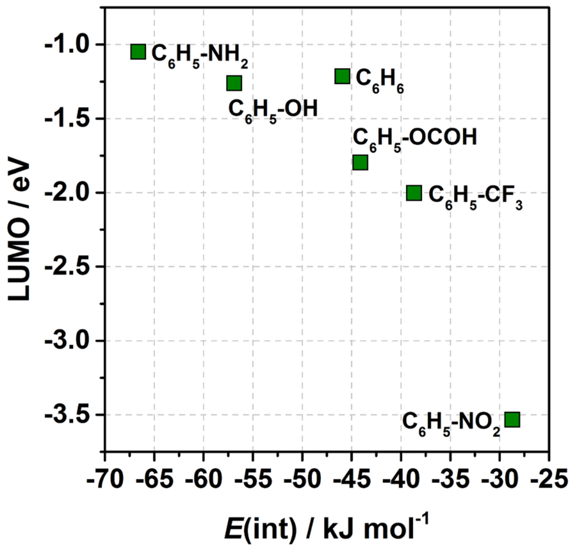

Figure S9. Correlation between energies of the LUMO and the DLPNO-CCSD(T) interaction energies of the Bi···π arene adducts.

Table S10. Sum of experimentally determined van der Waals radii[a] (in Å) of bismuth (r_{vdW} = 2.54 Å) and donor atoms (O, N, F, and Cl) of substituents.

Donor atom	r_{vdW}	Σr_{vdW}[b]
O	1.50	4.04
N	1.66	4.20
Cl	1.82	4.36
F	1.46	4.00

[a] S. Alvarez, *Dalton Trans.* **2013**, *42*, 8617.

[b] Σr_{vdW} – sum of van der Waals radii of bismuth and appropriate donor atom (O, N, F, or Cl).

Table S11. NBO partial charges (a.u.) for relaxed Bi···R adducts computed at the PBE-D3/def2-QZVP level of theory.

$BiCl_3$ adduct with	$\Sigma qBiCl_3$(adduct)	qBi (adduct)	ΔqBi	ΔqCl_3
NO_2	-0.070	1.303	0.029	-0.099
CN	-0.045	1.332	0.058	-0.103
CHO	-0.075	1.316	0.043	-0.118
F	-0.019	1.300	0.027	-0.046
Cl	-0.050	1.273	0.000	-0.051
OH	-0.042	1.303	0.029	-0.071
NH_2	-0.110	1.286	0.012	-0.122

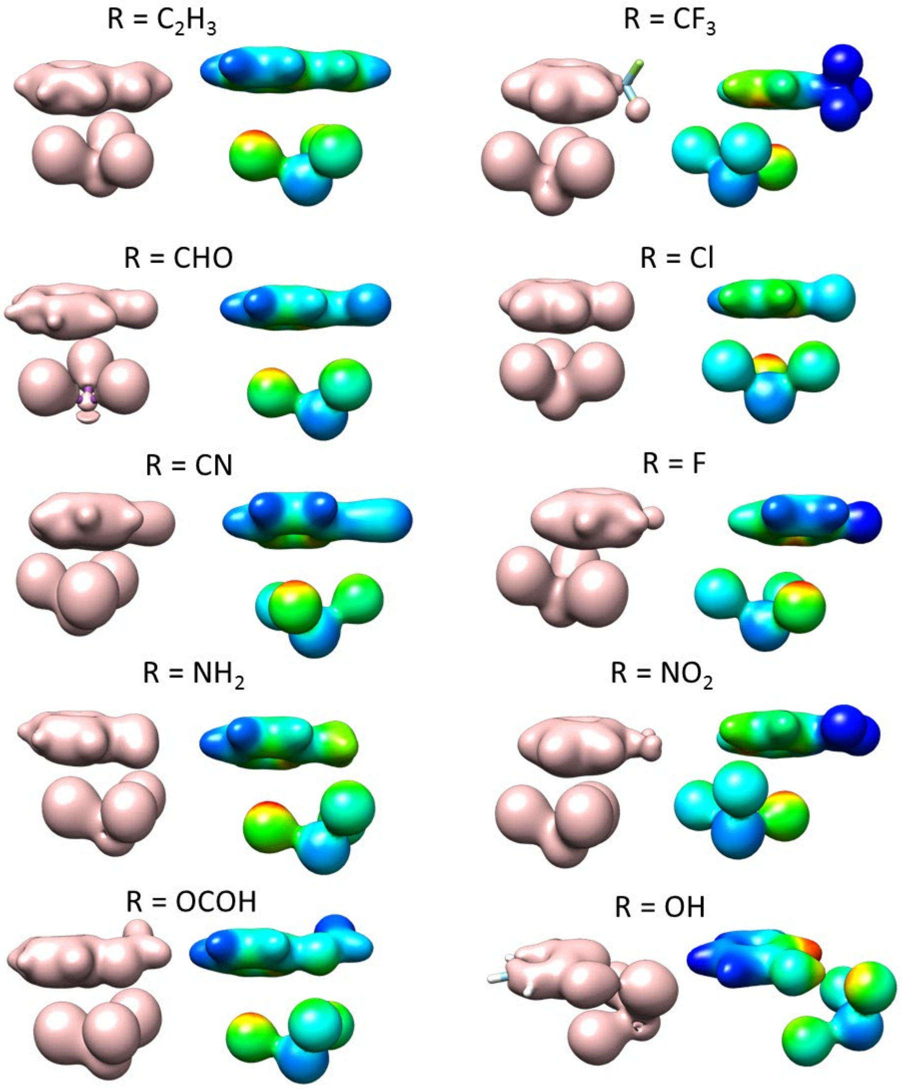

Figure S12. Dispersion energy plots for equilibrium structures of Cl···π arene adducts computed at the DLPNO-CCSD(T)/cc-pVQZ (cc-pwCVQZ-PP for bismuth) level of theory with tightPNO settings.

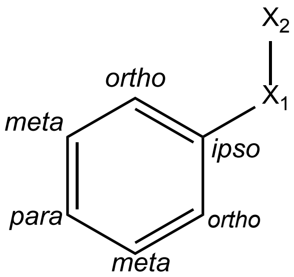

Scheme S13. Numbering of the atoms in NMR calculations.

Table S14. Δδ (ppm) values (gas phase) for selected benzene derivatives with one substituent computed using various density functionals with the pcsSeg-3 basis set.

**Bi···π**	Δδ *ipso*			Δδ *ortho*			Δδ *metha*			Δδ *para*			Δδ *X1*		
	TPSS	B3LYP	KT2	TPSS	B3LYP	KT2	TPSS	B3LYP	KT2	TPSS	B3LYP	KT2	TPSS	B3LYP	KT2
CHO	-1.25	-1.41	-1.40	-2.58	-2.67	-2.57	-3.82	-3.83	-3.82	-2.52	-3.10	-2.55	0.47	0.45	0.55
Cl	-7.01	-6.81	-6.51	-5.01	-5.08	-4.61	-2.89	-2.89	-3.09	0.15	-0.18	-0.36			
NO_2	-3.40	-3.41	-2.70	-4.28	-4.22	-3.77	-1.59	-1.19	-1.42	-1.30	-1.70	-0.82			
OH	-2.88	-2.67	-2.85	-4.75	-4.43	-4.80	-5.03	-4.80	-4.81	-5.78	-5.52	-4.91			
**Bi···R**	Δδ *ipso*			Δδ *ortho*			Δδ *metha*			Δδ *para*			Δδ *X1*		
	TPSS	B3LYP	KT2	TPSS	B3LYP	KT2	TPSS	B3LYP	KT2	TPSS	B3LYP	KT2	TPSS	B3LYP	KT2
CHO	2.43	3.43	2.37	-2.89	-3.19	-2.98	-0.90	-0.91	-0.88	-4.54	-5.16	-4.34	-4.74	-7.54	-4.00
Cl	5.29	5.77	4.88	-1.73	-2.00	-1.75	-0.53	-0.37	-0.64	-2.39	-2.76	-2.31			
NO_2	6.81	3.87	6.97	0.15	-0.51	0.39	-1.16	-0.89	-0.87	-1.57	-3.14	-0.74			
OH	5.64	6.25	5.45	-2.87	-2.54	-2.73	-0.92	-0.61	-0.78	-5.08	-4.92	-4.34			
**Cl···π**	Δδ *ipso*			Δδ *ortho*			Δδ *metha*			Δδ *para*			Δδ *X1*		
	TPSS	B3LYP	KT2	TPSS	B3LYP	KT2	TPSS	B3LYP	KT2	TPSS	B3LYP	KT2	TPSS	B3LYP	KT2
CHO	-0.25	-0.02	-0.71	-1.25	-1.33	-1.24	0.26	0.12	0.26	-0.67	-1.16	-0.71	-1.58	-1.81	-1.81
Cl	-0.04	-0.18	-0.09	-0.96	-1.03	-1.12	-1.35	-1.45	-1.49	0.40	0.31	0.47			
NO_2	0.59	0.52	0.82	-1.50	-1.40	-1.52	-1.05	-1.04	-0.89	-2.05	-2.26	-1.53			
OH	-0.05	-0.06	0.25	-1.84	-1.66	-2.01	-0.12	0.06	0.09	-0.19	-0.10	0.18			

Δδ=δ(adduct)- δ(free)

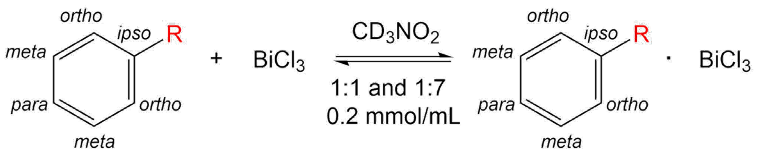

Table S15. ^{13}C NMR chemical shifts of the free monosubstituted arenes (C_6H_5R) and mixtures with $BiCl_3$ in a 1:1 and 1:7 molar ratio, measured in CD_3NO_2 solution at ambient temperature.

**δ-C_6H_5R**					
**^{13}C-NMR (CD_3NO_2)**	***δ-C_{ipso}***	***δ-C_{ortho}***	***δ-C_{meta}***	***δ-C_{para}***	***δ*-R**
C_6H_5F	164.25	116.37	131.61	125.70	-
C_6H_5F / $BiCl_3$ **(1:1)**	164.18	116.32	131.56	125.65	-
C_6H_5F / $BiCl_3$ **(1:7)**	163.79	116.07	131.31	125.40	-
C_6H_5Cl	135.13	129.77	131.41	128.17	-
C_6H_5Cl / $BiCl_3$ **(1:1)**	135.07	129.72	131.37	128.13	-
C_6H_5Cl / $BiCl_3$ **(1:7)**	134.71	129.44	131.12	128.87	-
$C_6H_5CF_3$	131.26	126.41	130.40	133.66	125.98
$C_6H_5CF_3$ / $BiCl_3$ **(1:1)**	131.19	126.35	130.35	133.61	125.91
$C_6H_5CF_3$ / $BiCl_3$ **(1:7)**	130.82	126.08	130.11	133.38	125.59
C_6H_5OH	157.74	116.48	130.98	121.53	-
C_6H_5OH / $BiCl_3$ **(1:1)**	157.61	116.47	130.96	121.56	-
C_6H_5OH / $BiCl_3$ **(1:7)**	156.93	116.46	130.82	121.62	-
C_6H_5CN	113.41	133.51	130.60	134.44	120.38
C_6H_5CN / $BiCl_3$ **(1:1)**	113.23	133.50	130.57	134.47	120.42
C_6H_5CN / $BiCl_3$ **(1:7)**	112.27	133.39	130.38	134.63	120.79
$C_6H_5NO_2$	149.61	124.61	130.86	136.33	-
$C_6H_5NO_2$ / $BiCl_3$ **(1:1)**	149.51	124.57	130.82	136.32	
$C_6H_5NO_2$ / $BiCl_3$ **(1:7)**	148.99	124.38	130.63	136.30	
$C_6H_5CH{=}CH_2$	139.05	127.47	129.97	129.26	114.64 (*CH_2*)<br>138.24(*CH*)
$C_6H_5CH{=}CH_2$ / $BiCl_3$ **(1:1)**	138.99	127.44	129.94	129.22	114.63 (*CH_2*)<br>138.19 (*CH*)
$C_6H_5CH{=}CH_2$ / $BiCl_3$ **(1:7)**	138.63	127.20	129.73	129.00	114.61 (*CH_2*)<br>137.82 (*CH*)
$C_6H_5CH{=}O$	138.05	130.81	130.43	135.87	194.53
$C_6H_5CH{=}O$ / $BiCl_3$ **(1:1)**	137.81	131.00	130.44	136.13	195.32
$C_6H_5CH{=}O$ / $BiCl_3$ **(1:7)**	136.85	131.57	130.36	136.96	197.91

Low-field ⟶ High-field

Table S16. The difference between the chemical shift of the arene in the mixture with $BiCl_3$ and the chemical shift of the free monosubstituted arene.

**$\Delta\delta = \delta$(adduct with $BiCl_3$) – δ(free)**					
**^{13}C-NMR (CD_3NO_2)**	***$\Delta\delta$-C_{ipso}***	***$\Delta\delta$-C_{ortho}***	***$\Delta\delta$-C_{meta}***	***$\Delta\delta$-C_{para}***	***$\Delta\delta$-R***
C_6H_5F / $BiCl_3$ **(1:1)**	-0.07	-0.05	-0.05	-0.05	-
C_6H_5F / $BiCl_3$ **(1:7)**	-0.46	-0.33	-0.30	-0.30	-
C_6H_5Cl / $BiCl_3$ **(1:1)**	-0.06	-0.05	-0.04	-0.04	-
C_6H_5Cl / $BiCl_3$ **(1:7)**	-0.42	-0.33	-0.29	0.70	-
$C_6H_5CF_3$ / $BiCl_3$ **(1:1)**	-0.07	-0.06	-0.05	-0.05	-0.07
$C_6H_5CF_3$ / $BiCl_3$ **(1:7)**	-0.44	-0.33	-0.29	-0.28	-0.39
C_6H_5OH / $BiCl_3$ **(1:1)**	-0.13	-0.01	-0.02	0.03	-
C_6H_5OH / $BiCl_3$ **(1:7)**	-0.81	-0.02	-0.16	0.09	-
C_6H_5CN / $BiCl_3$ **(1:1)**	-0.18	-0.01	-0.03	0.03	0.04
$C_6H_5NO_2$ / $BiCl_3$ **(1:1)**	-0.1	-0.04	-0.04	-0.01	
$C_6H_5NO_2$ / $BiCl_3$ **(1:7)**	-0.62	-0.23	-0.23	-0.03	
$C_6H_5CH{=}CH_2$ / $BiCl_3$ **(1:1)**	-0.06	-0.03	-0.03	-0.04	-0.01 (CH_2)<br>-0.05 (CH)
$C_6H_5CH{=}CH_2$ / $BiCl_3$ **(1:7)**	-0.42	-0.27	-0.24	-0.26	-0.03 (CH_2)<br>-0.42 (CH)
$C_6H_5CH{=}O$ / $BiCl_3$ **(1:1)**	-0.24	0.19	0.01	0.26	0.79
$C_6H_5CH{=}O$ / $BiCl_3$ **(1:7)**	-1.20	0.76	-0.07	1.09	3.38

Low-field ⟶ High-field

^{13}C NMR studies in solution were carried out in order to investigated the interaction between dispersion energy donors i. e. $BiCl_3$ and a series of monosubstituted arene ligands in a 1:1 and 7:1 molar ratio, in CD_3NO_2 solution. The experimental results of the solution ^{13}C NMR spectroscopy show changes for all analyzed systems and in almost all cases a high-field shift is observed for the chemical shifts of the arene in the mixture with $BiCl_3$ compared to the respective free monosubstituted arene. Larger chemical shifts are observed as the content of $BiCl_3$ is increased. In contrast to the *ipso*, *ortho* and *meta* carbons the *para* carbon shows only minor changes of the chemical shift and for about half of the analyzed systems a low-field shift is observed. In the case of the assummed benzaldehyde·$BiCl_3$ adduct larger shifts were observed for the C atoms, but this most likely is due to the fact that the benzaldehyde preferentially coordinates over the O atom and not to the π system, implying that oxygen is a better donor than the π system. This is in accordance with the theoretical calculations as discussed and given in Table 6. The chemical shifts observed from the experiment are significantly smaller than those obtained from the theoretical calculations and a trend with regard to the substituents as found in the theoretical part, does not become obvious. Most likely, this is because we have to consider certain equilibria and interference with the polar solvent nitromethane (see Figure S4).

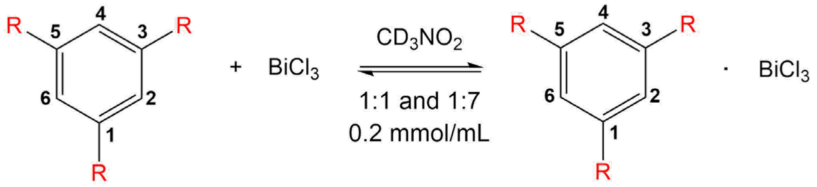

Table S17. ^{13}C NMR chemical shifts of the free trisubstituted arenes ($C_6H_3R_3$-1,3,5) and mixtures with $BiCl_3$ and in a 1:1 and 1:7 molar ratio, measured in CD_3NO_2 solution at ambient temperature. The difference between the chemical shift of the $BiCl_3$ adduct and the chemical shift of the free trisubstituted arene.

	**δ-$C_6H_3R_3$-1,3,5**			**$\Delta\delta$ =** δ(adduct with $BiCl_3$) – δ(free)		
**^{13}C-NMR (CD_3NO_2)**	**δ C_1,C_3,C_5**	**δ C_2,C_4,C_6**	**δ-R**	**$\Delta\delta$ C_1,C_3,C_5**	**$\Delta\delta$ C_2,C_4,C_6**	**$\Delta\delta$-R**
1,3,5-$C_6H_3F_3$	164.71	101.46	-	-	-	-
1,3,5-$C_6H_3F_3$ / $BiCl_3$ **(1:1)**	164.64	101.41	-	-0.07	-0.05	-
1,3,5-$C_6H_3F_3$ / $BiCl_3$ **(1:7)**	164.27	101.17	-	-0.44	-0.29	-
1,3,5-$C_6H_3Cl_3$	136.70	128.51	-	-	-	-
1,3,5-$C_6H_3Cl_3$ / $BiCl_3$ **(1:1)**	136.64	128.45	-	-0.06	-0.06	-
1,3,5-$C_6H_3Cl_3$ / $BiCl_3$ **(1:7)**	136.29	128.16	-	-0.41	-0.35	-
1,3,5-$C_6H_3(CF_3)_3$	133.56	127.72	124.34	-	-	-
1,3,5-$C_6H_3(CF_3)_3$ / $BiCl_3$ **(1:1)**	133.50	127.66	124.28	-0.06	-0.06	-0.06
1,3,5-$C_6H_3(CF_3)_3$ / $BiCl_3$ **(1:7)**	133.15	127.34	123.93	-0.41	-0.38	-0.41
1,3,5-$C_6H_3(CN)_3$	116.26	140.92	116.96	-	-	-
1,3,5-$C_6H_3(CN)_3$ / $BiCl_3$ **(1:1)**	116.19	140.91	116.93	-0.07	-0.01	-0.03
1,3,5-$C_6H_3(CN)_3$ / $BiCl_3$ **(1:7)**	115.89	140.88	116.84	-0.37	-0.04	-0.12

Low-field ⟶ High-field

Table S18. Δδ values for nitrobenzene computed at the M06L/pcSseg-3 level of theory in the gas phase, with CPCM solvation model and with explicit solvent molecules.

Position	Gas phase	CPCM[a]	Explicit solvent[b]
ipso	-3.86	-3.69	0.21
ortho	-3.98	-3.73	-3.55
meta	-0.61	-1.32	-2.98
para	-0.77	0.76	6.22

[a] $\Delta\delta^{CPCM}=\delta(\text{adduct})^{CPCM}-\delta(\text{free})^{CPCM}$

[b] $\Delta\delta^{explicit}=\delta(\text{nitrobenzene})^{explicit\ solvent}-\delta(\text{nitrobenzene})^{gas\ phase}$

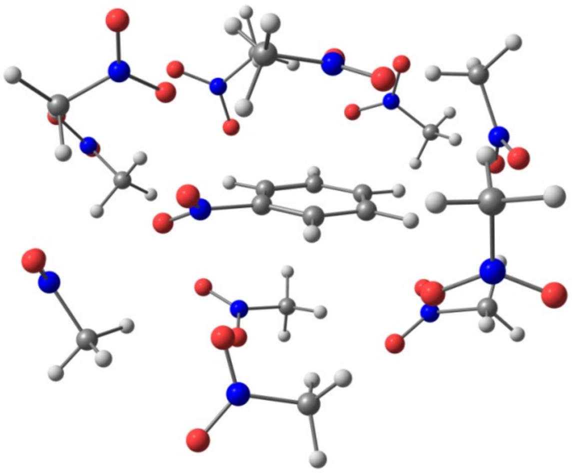

Figure S19. Nitrobenzene molecule surrounded by explicit nitromethane molecules optimized at the PBE-D3/def2-SVP level of theory.

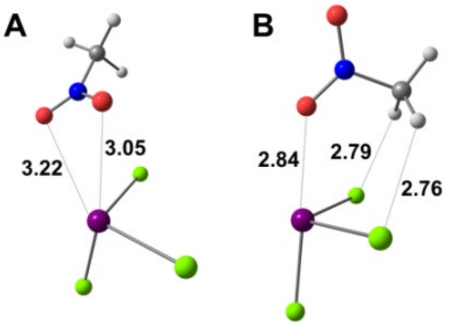

Figure S20. Possible motifs of interaction between solvent and $BiCl_3$ molecule as calculated at the PBE-D3/def2-QZVP level of theory. The interaction energy for structure A amounts to -41 kJ mol^{-1} while the interaction energy for structure B is -46 kJ mol^{-1}.

In order to assess the influence of the solvation on the computed NMR spectra we have chosen nitrobenzene and nitrobenzene adduct with the $BiCl_3$ molecule and used the CPCM solvation model with acetonitrile as a solvent due to the fact that its dielectric constant (ε=36.6) is similar to the one of nitromethane. Additionally, we examined the influence of explicit solvation on the ^{13}C Δδ values for free nitrobenzene. Therefore, we optimized nitrobenzene surrounded by eleven nitromethane molecules as a first solvation shell at the PBE-D3/def2-SVP level of theory. The obtained ^{13}C δ values were compared to the ^{13}C NMR chemical shifts computed for nitrobenzene in gas phase. The structure of the system is shown in Figure S5 and the results both for CPCM as well as for explicit solvation are shown in Table S12. The results were compared to the ^{13}C Δδ values from the gas phase calculations. The CPCM model gives similar trend as the gas phase calculations for predicting the carbon chemical shifts. An estimate of the solvent effects using explicit molecules yields similar trends but also shows that for a quantitative agreement long MD simulations with snapshot NMR calculations would be necessary, which goes beyond the scope of the current investigation.

Experimental details of NMR measurements

^{1}H and $^{13}C\{^{1}H\}$ NMR spectra were recorded at ambient temperature in CD_3NO_2 with an *Avance III 500* spectrometer (Bruker) at 500.30 and 125.81 MHz, respectively, and are referenced internally to the deuterated solvent relative to $Si(CH_3)_4$ ($\delta = 0.00$ ppm). The NMR spectra were processed using the software *MestReNova* (version 11.0.0-17609[1] / version 11.0.4-18998[2]).

Refrences:

1. *MestReNova*, (version 11.0.0-17609), Mestrelab Research S. L., Santiago de Compostela, **2016**.
2. *MestReNova*, (version 11.0.4-18998), Mestrelab Research S. L., Santiago de Compostela, **2017**.

Cartesian coordinates

Substrates

$BiCl_3$

Bi	0.001074000	0.000000000	0.394379000
Cl	2.151045000	0.000000000	-0.786835000
Cl	-1.076060000	1.860369000	-0.787428000
Cl	-1.076060000	-1.860369000	-0.787428000

$Bi(CH_3)_3$

Bi	0.000601000	0.000000000	0.227451000
C	-0.953513000	1.649782000	-1.023849000
C	-0.953512000	-1.649783000	-1.023849000
C	1.907159000	0.000000000	-1.022302000
H	-0.469763000	2.605322000	-0.793492000
H	-0.818903000	1.407201000	-2.083437000
H	-2.021208000	1.711915000	-0.786470000
H	-2.021208000	-1.711914000	-0.786472000
H	-0.818901000	-1.407202000	-2.083437000
H	-0.469763000	-2.605322000	-0.793491000
H	2.493548000	-0.895056000	-0.787314000
H	1.631914000	0.000001000	-2.082414000
H	2.493548000	0.895056000	-0.787314000

$AsCl_3$

As	-0.000313867	0.000000565	-0.602890851
Cl	-0.968839221	-1.679266871	0.430445273
Cl	-0.968840172	1.679267533	0.430443964
Cl	1.937993261	-0.000000228	0.430620615

$SbCl_3$

Sb	-0.000267065	0.000000001	-0.537017208
Cl	2.060488455	0.000000015	0.618880901
Cl	-1.030111209	-1.783499332	0.618731154
Cl	-1.030111182	1.783499316	0.618731153

Ethenylbenzene (C_2H_3-C_6H_5)

C	-0.085483000	0.009150000	-5.610996000
C	-0.832268000	-0.950131000	-4.918449000
C	0.658944000	0.947390000	-4.895718000
H	-1.416716000	-1.686530000	-5.470536000
H	1.244234000	1.700097000	-5.424111000
C	-0.834879000	-0.971552000	-3.527597000
C	0.655063000	0.924189000	-3.501571000
H	-1.423843000	-1.726240000	-3.006446000
H	1.238436000	1.659432000	-2.944389000
C	-0.089310000	-0.033023000	-2.789591000
H	-0.086345000	0.022606000	-6.700756000
C	-0.055516000	-0.010186000	-1.323515000
H	0.563050000	0.785050000	-0.896480000
C	-0.686456000	-0.840812000	-0.480544000
H	-1.320237000	-1.658126000	-0.825537000
H	-0.585352000	-0.725211000	0.597171000

Trifluoromethylbenzene (CF_3-C_6H_5)

C	-0.087835000	0.013597000	-5.609851000
C	-1.242930000	-0.341825000	-4.910225000
C	1.074814000	0.348911000	-4.913767000
H	-2.150679000	-0.607149000	-5.451260000
H	1.978362000	0.623083000	-5.457417000
C	-1.238333000	-0.361853000	-3.516351000
C	1.085976000	0.331258000	-3.519266000
H	-2.134623000	-0.643403000	-2.965640000
H	1.991235000	0.587187000	-2.971210000
C	-0.071703000	-0.025950000	-2.822560000
H	-0.093338000	0.026535000	-6.699597000
C	-0.083781000	-0.000558000	-1.316553000
F	-0.528586000	1.199708000	-0.838725000
F	-0.902639000	-0.952412000	-0.793617000
F	1.151671000	-0.197129000	-0.785459000

Benzaldehyde (CHO-C_6H_5)

C	-0.081459000	0.002935000	-5.602793000
C	-0.805423000	-0.976230000	-4.910543000
C	0.637953000	0.975466000	-4.904551000
H	-1.363623000	-1.731167000	-5.464097000
H	1.199568000	1.735197000	-5.447566000
C	-0.811311000	-0.984583000	-3.520387000
C	0.632503000	0.968042000	-3.510688000
H	-1.365236000	-1.734275000	-2.955146000
H	1.189539000	1.721250000	-2.949409000
C	-0.090278000	-0.009420000	-2.813185000
H	-0.079609000	0.005808000	-6.692974000
C	-0.081846000	0.002401000	-1.334368000
O	-0.668972000	-0.794207000	-0.624056000
H	0.527135000	0.829383000	-0.886159000

Chlorobenzene (Cl-C_6H_5)

C	-0.083492000	0.000000000	-5.619348000
C	-1.237736000	-0.354174000	-4.918280000
C	1.070751000	0.354174000	-4.918280000
H	-2.143261000	-0.631982000	-5.457447000
H	1.976276000	0.631982000	-5.457447000
C	-1.245902000	-0.356669000	-3.522660000
C	1.078917000	0.356669000	-3.522660000
H	-2.140423000	-0.631008000	-2.966366000
H	1.973438000	0.631008000	-2.966366000
C	-0.083492000	0.000000000	-2.838123000
Cl	-0.083492000	0.000000000	-1.094872000
H	-0.083493000	0.000000000	-6.708725000

Benzonitrile (CN-C_6H_5)

C	-0.083492000	0.000000000	-5.599673000
C	-1.240891000	-0.355142000	-4.902504000
C	1.073906000	0.355142000	-4.902504000
H	-2.143517000	-0.632048000	-5.446105000
H	1.976532000	0.632048000	-5.446105000
C	-1.247807000	-0.357251000	-3.510421000
C	1.080822000	0.357251000	-3.510421000

Fluorobenzene (F-C_6H_5)

C	-0.083493000	0.000000000	-5.377494000
C	-1.238894000	-0.354520000	-4.677574000
C	1.071909000	0.354520000	-4.677574000
H	-2.143178000	-0.631931000	-5.218803000
H	1.976193000	0.631931000	-5.218803000
C	-1.248394000	-0.357439000	-3.281157000
C	1.081409000	0.357439000	-3.281157000

H	-2.144254000	-0.632198000	-2.957015000
H	1.977269000	0.632198000	-2.957015000
C	-0.083493000	0.000000000	-2.807745000
H	-0.083493000	0.000000000	-6.689367000
C	-0.083492000	0.000000000	-1.379140000
N	-0.083492000	0.000000000	-0.214079000

H	-2.136511000	-0.629836000	-2.713175000
H	1.969526000	0.629836000	-2.713175000
C	-0.083493000	0.000000000	-2.611634000
F	-0.083492000	0.000000000	-1.253300000
H	-0.083493000	0.000000000	-6.466728000

Aniline (NH_2-C_6H_5)

C	-0.089667000	-0.008614000	-5.619061000
C	-1.072803000	0.682023000	-4.906382000
C	0.908634000	-0.682840000	-4.911759000
H	-1.859691000	1.216059000	-5.439841000
H	1.686064000	-1.226319000	-5.449537000
C	-1.062740000	0.700693000	-3.513616000
C	0.927773000	-0.670446000	-3.518985000
H	-1.839166000	1.240368000	-2.968062000
H	1.711025000	-1.204800000	-2.978009000
C	-0.059856000	0.023390000	-2.797754000
H	-0.100425000	-0.019908000	-6.707952000
N	-0.081129000	-0.012197000	-1.402978000
H	-0.575586000	0.752930000	-0.957291000
H	0.811467000	-0.199223000	-0.959406000

Nitrobenzene (NO_2-C_6H_5)

C	-0.083491000	-0.000001000	-5.612358000
C	-0.811744000	-0.969195000	-4.916986000
C	0.644760000	0.969194000	-4.916986000
H	-1.378484000	-1.723567000	-5.461358000
H	1.211502000	1.723565000	-5.461358000
C	-0.816347000	-0.975740000	-3.523537000
C	0.649356000	0.975744000	-3.523537000
H	-1.371312000	-1.715001000	-2.950704000
H	1.204320000	1.715006000	-2.950703000
C	-0.083497000	0.000003000	-2.850830000
H	-0.083489000	-0.000003000	-6.702133000
N	-0.083496000	0.000003000	-1.368483000
O	-0.734700000	-0.877461000	-0.798898000
O	0.567728000	0.877451000	-0.798898000

Oxophenoxymethyl (OCHO-C_6H_5)

C	-0.036150000	-0.048670000	-5.595787000
C	-1.004419000	-0.824806000	-4.955451000
C	0.805475000	0.771600000	-4.842568000
H	-1.664867000	-1.465641000	-5.539038000
H	1.561379000	1.382638000	-5.335107000
C	-1.138473000	-0.790120000	-3.566962000
C	0.684601000	0.814340000	-3.453071000
H	-1.888716000	-1.394354000	-3.062440000
H	1.328684000	1.448141000	-2.845111000
C	-0.284930000	0.030998000	-2.833037000
H	0.060739000	-0.082090000	-6.680385000
O	-0.411365000	0.173999000	-1.440376000
C	-0.416375000	-0.937958000	-0.647866000
O	-0.302589000	-2.081670000	-1.002464000
H	-0.538511000	-0.596285000	0.396868000

Phenol (OH-C_6H_5)

C	-0.085365000	0.001336000	-5.617150000
C	-1.056987000	0.719803000	-4.918991000
C	0.881191000	-0.713222000	-4.903840000
H	-1.817749000	1.281912000	-5.460778000
H	1.645467000	-1.278129000	-5.437835000
C	-1.065957000	0.726597000	-3.523498000
C	0.883429000	-0.714718000	-3.510499000
H	-1.827994000	1.289589000	-2.979511000
H	1.632117000	-1.268021000	-2.944833000
C	-0.094102000	0.008058000	-2.817948000
H	-0.080845000	-0.002182000	-6.706189000
O	-0.051746000	-0.023253000	-1.447447000
H	-0.781454000	0.515239000	-1.102392000

1,3,5-triethenylbenzene

C	-0.045620000	0.017693000	-5.603270000
C	-0.821937000	-0.954131000	-4.944428000
C	0.708342000	0.958217000	-4.894058000
C	-0.824854000	-0.966075000	-3.545922000
C	0.682844000	0.912652000	-3.487527000
H	-1.425144000	-1.718590000	-3.032944000
H	1.268577000	1.629845000	-2.911016000
C	-0.075683000	-0.038620000	-2.797852000
H	-0.025345000	0.049227000	-6.693412000
C	-0.056396000	-0.030169000	-1.330472000
H	0.572839000	0.748731000	-0.889672000
C	-0.713286000	-0.856033000	-0.503400000
H	-1.359999000	-1.656803000	-0.862632000
H	-0.621680000	-0.752945000	0.576435000
C	-1.624403000	-1.952096000	-5.661372000
H	-2.169404000	-2.645448000	-5.013883000
C	-1.751037000	-2.092959000	-6.988673000
H	-1.242143000	-1.442183000	-7.699989000
H	-2.378681000	-2.876606000	-7.409372000
C	1.490474000	1.948695000	-5.643119000
H	1.453943000	1.828147000	-6.729912000
C	2.216363000	2.958410000	-5.141924000
H	2.296022000	3.150075000	-4.071630000
H	2.758306000	3.636278000	-5.799067000

1,3,5-tri(trifluoromethyl)benzene

C	-0.103755000	0.002254000	-5.616007000
C	-1.253047000	-0.343884000	-4.897385000
C	1.059060000	0.332786000	-4.927582000
C	-1.241196000	-0.358021000	-3.506150000
C	1.084701000	0.321909000	-3.529116000
H	-2.136189000	-0.633829000	-2.952648000
H	1.998831000	0.574388000	-2.994624000
C	-0.065334000	-0.024505000	-2.826108000
H	-0.116666000	0.008346000	-6.704281000
C	-0.068274000	-0.001110000	-1.315211000
F	-0.574315000	1.172486000	-0.844374000
F	-0.832661000	-0.996986000	-0.799573000
F	1.178510000	-0.133734000	-0.800773000
C	-2.503536000	-0.698632000	-5.667620000
F	-2.923889000	0.347346000	-6.429594000
F	-2.282154000	-1.738742000	-6.515642000
F	-3.529282000	-1.047223000	-4.856070000
C	2.316052000	0.722747000	-5.669866000
F	2.647070000	2.020073000	-5.426151000
F	3.375983000	-0.033955000	-5.279153000
F	2.188084000	0.582767000	-7.010357000

1,3,5-trichlorobenzene

1,3,5-Benzenetricarboxaldehyde

C	-0.083492000	0.000000000	-5.630421000
C	-1.226950000	-0.350856000	-4.912467000
C	1.059966000	0.350856000	-4.912467000
C	-1.249677000	-0.357919000	-3.517680000
C	1.082692000	0.357919000	-3.517681000
H	-2.149290000	-0.634034000	-2.974130000
H	1.982306000	0.634034000	-2.974130000
C	-0.083492000	0.000000000	-2.840932000
Cl	-0.083492000	0.000000000	-1.103790000
H	-0.083493000	0.000000000	-6.717152000
Cl	-2.665029000	-0.791780000	-5.781314000
Cl	2.498044000	0.791780000	-5.781314000

C	-0.076352000	0.010065000	-5.617215000
C	-0.804133000	-0.973010000	-4.925051000
C	0.641372000	0.978940000	-4.917244000
C	-0.806135000	-0.975851000	-3.530807000
C	0.634261000	0.969197000	-3.511864000
H	-1.364353000	-1.730242000	-2.971649000
H	1.201840000	1.735119000	-2.978433000
C	-0.085461000	-0.002471000	-2.817773000
H	-0.085436000	-0.001986000	-6.709520000
C	-0.086042000	-0.002889000	-1.332628000
O	-0.684084000	-0.811441000	-0.649055000
H	0.518657000	0.815404000	-0.868863000
C	-1.568692000	-2.006756000	-5.668179000
O	-1.622356000	-2.078080000	-6.880919000
H	-2.109309000	-2.739147000	-5.018847000
C	1.406620000	2.013008000	-5.659271000
O	2.057744000	2.893205000	-5.130123000
H	1.342876000	1.927020000	-6.772303000

1,3,5-Benzenetricarbonitrile

C	-0.083493000	0.000000000	-5.623585000
C	-1.241922000	-0.355452000	-4.917963000
C	1.074937000	0.355452000	-4.917963000
C	-1.246960000	-0.357015000	-3.515754000
C	1.079975000	0.357014000	-3.515754000
H	-2.147619000	-0.633312000	-2.971733000
H	1.980634000	0.633311000	-2.971733000
C	-0.083492000	0.000000000	-2.819234000
H	-0.083493000	0.000000000	-6.711476000
C	-0.083492000	0.000000000	-1.389740000
N	-0.083492000	0.000000000	-0.225831000
C	-2.425499000	-0.718521000	-5.632621000
N	-3.389325000	-1.014197000	-6.214227000
C	2.258514000	0.718522000	-5.632621000
N	3.222340000	1.014197000	-6.214228000

1,3,5-trifluorobenzene

C	-0.083493000	0.000000000	-5.394239000
C	-1.216278000	-0.347577000	-4.665639000
C	1.049293000	0.347577000	-4.665639000
C	-1.253226000	-0.358922000	-3.275155000
C	1.086241000	0.358922000	-3.275155000
H	-2.153058000	-0.634941000	-2.731622000
H	1.986073000	0.634941000	-2.731622000
C	-0.083493000	0.000000000	-2.613343000
F	-0.083492000	0.000000000	-1.261399000
H	-0.083493000	0.000000000	-6.481133000
F	-2.335522000	-0.690911000	-5.341951000
F	2.168536000	0.690911000	-5.341951000

1,3,5-Benzenetriamine

C	-0.063100000	0.030696000	-5.617842000
C	-1.062081000	0.717595000	-4.913832000
C	0.937839000	-0.659370000	-4.919610000
C	-1.057017000	0.719327000	-3.511880000
C	0.943217000	-0.657838000	-3.517660000
H	-1.843242000	1.242105000	-2.963948000
H	1.714305000	-1.206971000	-2.974145000
C	-0.056058000	0.029037000	-2.813920000
H	-0.075372000	0.017318000	-6.709313000
N	-0.088217000	-0.022940000	-1.416649000
H	-0.576176000	0.748108000	-0.973562000
H	0.807717000	-0.202772000	-0.976348000
N	-2.096689000	1.352151000	-5.609088000
H	-1.865387000	1.627358000	-6.557640000
H	-2.551545000	2.103092000	-5.101001000
N	1.896613000	-1.397494000	-5.621216000
H	2.762705000	-1.556913000	-5.117766000
H	2.067012000	-1.080101000	-6.569483000

1,3,5-trinitrobenzene

C	-0.083905000	0.000312000	-5.628694000
C	-0.798304000	-0.949338000	-4.906413000
C	0.630672000	0.949826000	-4.906412000
C	-0.816767000	-0.974702000	-3.516109000
C	0.649554000	0.974875000	-3.516108000
H	-1.382589000	-1.727283000	-2.972633000
H	1.215521000	1.727346000	-2.972630000
C	-0.083510000	0.000013000	-2.848229000
H	-0.084070000	0.000437000	-6.715866000
N	-0.083291000	-0.000152000	-1.357385000
O	-0.734658000	-0.880008000	-0.800480000
O	0.567753000	0.879940000	-0.800476000
N	-1.574441000	-1.981109000	-5.651862000
O	-2.195057000	-2.802760000	-4.982342000
O	-1.531187000	-1.931973000	-6.878354000
N	1.406514000	1.981821000	-5.651859000
O	1.365234000	1.931194000	-6.878359000
O	2.029676000	2.801551000	-4.982350000

1,3,5-triformate 1,3,5-Benzenetriol

C	0.101385000	-0.093843000	-5.557016000
C	-0.734025000	-1.015730000	-4.930868000
C	0.955674000	0.659425000	-4.755760000
C	-0.748338000	-1.198105000	-3.549700000
C	0.977829000	0.513257000	-3.371723000
H	-1.408623000	-1.920288000	-3.083341000
H	1.648244000	1.110702000	-2.758318000
C	0.118574000	-0.414996000	-2.791737000
H	0.078815000	0.015658000	-6.636728000

1,3,5-Benzenetriol

C	-0.085365000	0.001336000	-5.617150000
C	-1.056987000	0.719803000	-4.918991000
C	0.881191000	-0.713222000	-4.903840000
H	-1.817749000	1.281912000	-5.460778000
H	1.645467000	-1.278129000	-5.437835000
C	-1.065957000	0.726597000	-3.523498000
C	0.883429000	-0.714718000	-3.510499000
H	-1.827994000	1.289589000	-2.979511000
H	1.632117000	-1.268021000	-2.944833000

O	0.230902000	-0.573962000	-1.405663000	C	-0.094102000	0.008058000	-2.817948000
C	-0.897818000	-0.577234000	-0.629694000	H	-0.080845000	-0.002182000	-6.706189000
O	-2.029846000	-0.425978000	-1.002797000	O	-0.051746000	-0.023253000	-1.447447000
H	-0.574870000	-0.737815000	0.414972000	H	-0.781454000	0.515239000	-1.102392000
O	-1.619583000	-1.697076000	-5.772870000				
C	-1.775833000	-3.052204000	-5.649212000				
O	-1.207805000	-3.777278000	-4.877791000				
H	-2.523136000	-3.363299000	-6.401597000				
O	1.783592000	1.660407000	-5.275165000				
C	2.531273000	1.424717000	-6.398100000				
O	2.586144000	0.404348000	-7.030114000				
H	3.088410000	2.353769000	-6.617607000				

Equilibrium structures

Bi···π arene adducts

C_2H_3-C_6H_5···$BiCl_3$				CF_3-C_6H_5···$BiCl_3$			
Bi	3.105283000	-0.212870000	0.076484000	C	-0.052849000	0.346524000	-5.532230000
C	0.201915000	1.366193000	0.109484000	C	-1.174284000	-0.271020000	-4.968819000
C	0.031138000	0.781657000	-1.152424000	C	1.050836000	0.654299000	-4.731724000
C	-0.205779000	-0.592134000	-1.245475000	H	-2.034939000	-0.510848000	-5.591854000
C	-0.263076000	-1.374007000	-0.089510000	H	1.921227000	1.146003000	-5.163513000
C	-0.074573000	-0.807395000	1.185929000	C	-1.196752000	-0.575905000	-3.607300000
C	0.148414000	0.583827000	1.263546000	C	1.034998000	0.351162000	-3.367415000
C	-0.096652000	-1.673776000	2.369918000	H	-2.067077000	-1.057212000	-3.163425000
C	0.398685000	-1.371408000	3.578018000	H	1.888354000	0.600559000	-2.739144000
H	0.892006000	-0.422170000	3.786994000	C	-0.090930000	-0.261474000	-2.807863000
H	0.349854000	-2.087531000	4.396356000	H	-0.045009000	0.600530000	-6.591023000
H	-0.532842000	-2.663852000	2.211154000	C	-0.153400000	-0.503877000	-1.320552000
H	0.277332000	1.054894000	2.236958000	F	-0.724785000	0.566685000	-0.683314000
H	0.385508000	2.437013000	0.191238000	F	-0.902773000	-1.589433000	-1.007299000
H	0.082492000	1.395563000	-2.050494000	F	1.072105000	-0.678344000	-0.772656000
H	-0.347653000	-1.057168000	-2.220892000	Bi	-1.050382000	3.109708000	-3.611228000
H	-0.447152000	-2.446541000	-0.169189000	Cl	-2.439151000	4.819490000	-2.508009000
Cl	5.055709000	-1.625978000	-0.502518000	Cl	-1.462534000	3.784506000	-5.950463000
Cl	3.728482000	0.320668000	2.412375000	Cl	1.148920000	4.187543000	-3.310099000
Cl	3.869243000	1.892847000	-0.970205000				

CHO-C_6H_5···$BiCl_3$				Cl-C6H5···BiCl3			
Bi	3.191658000	0.288559000	0.068819000	Bi	3.180642000	-0.138837000	0.166942000
C	-0.301320000	1.347561000	-0.045026000	C	0.229486000	1.304707000	-0.590394000
C	-0.099406000	0.447492000	-1.103911000	C	0.363033000	0.350847000	-1.604202000
C	0.156575000	-0.905696000	-0.833094000	C	0.149529000	-1.005061000	-1.335254000
C	0.219008000	-1.357465000	0.486956000	C	-0.201562000	-1.395454000	-0.040081000
C	0.019307000	-0.455496000	1.538081000	C	-0.338046000	-0.452831000	0.984801000
C	-0.242595000	0.895165000	1.271505000	C	-0.121342000	0.897573000	0.701519000
C	-0.133890000	0.919008000	-2.512666000	Cl	-0.460619000	-3.077697000	0.306783000
O	-0.333525000	2.072596000	-2.842407000	H	-0.610835000	-0.779142000	1.986906000
H	0.048475000	0.120701000	-3.273094000	H	-0.218997000	1.634343000	1.498103000
H	0.327083000	-1.593740000	-1.662642000	H	0.410922000	2.357198000	-0.801189000
H	0.436633000	-2.403197000	0.699206000	H	0.651442000	0.654985000	-2.609505000
H	0.075264000	-0.806168000	2.568274000	H	0.260392000	-1.753671000	-2.117561000
H	-0.399362000	1.590645000	2.095797000	Cl	5.085166000	-1.190373000	1.334850000
H	-0.499804000	2.392977000	-0.282909000	Cl	3.722766000	2.221889000	0.653053000
Cl	3.549476000	-0.661772000	-2.182482000	Cl	4.070566000	-0.246917000	-2.133696000
Cl	3.885823000	-1.667827000	1.405524000				
Cl	5.277633000	1.590993000	0.228756000				

CN-C_6H_5···$BiCl_3$				F-C_6H_5···$BiCl_3$			
Bi	3.330781000	-0.170976000	-0.271327000	Bi	3.200139000	-0.098546000	0.219251000
C	0.161883000	1.250676000	-0.151930000	C	0.174890000	1.289779000	0.273236000
C	-0.094901000	0.504284000	-1.307493000	C	0.233413000	0.825482000	-1.045373000
C	-0.224589000	-0.881002000	-1.234301000	C	0.065341000	-0.534843000	-1.326715000
C	-0.095748000	-1.529489000	0.009195000	C	-0.165362000	-1.407777000	-0.266858000
C	0.164392000	-0.779204000	1.170784000	C	-0.224049000	-0.974008000	1.056470000
C	0.291690000	0.607457000	1.083453000	C	-0.053289000	0.387846000	1.319314000

C	-0.217229000	-2.951070000	0.087869000
N	-0.308332000	-4.110413000	0.150645000
H	0.274643000	-1.287735000	2.126979000
H	0.507857000	1.184607000	1.981165000
H	0.277728000	2.331305000	-0.216750000
H	-0.190340000	1.005641000	-2.269790000
H	-0.425352000	-1.470763000	-2.127328000
Cl	3.878173000	-0.067080000	2.129932000
Cl	3.659689000	2.212909000	-0.815755000
Cl	5.491349000	-1.014054000	-1.096279000

F	-0.332311000	-2.723750000	-0.528496000
H	-0.403232000	-1.694425000	1.853046000
H	-0.090344000	0.745149000	2.347712000
H	0.318506000	2.347347000	0.487250000
H	0.426717000	1.519418000	-1.862164000
H	0.113638000	-0.918409000	-2.344452000
Cl	5.195013000	-1.442285000	0.788640000
Cl	3.726856000	1.889713000	1.586765000
Cl	3.951162000	0.769012000	-1.968022000

NH_2-C_6H_5···$BiCl_3$

C	-0.783831000	-0.193580000	-5.513046000
C	-1.399951000	0.498845000	-4.464801000
C	0.484884000	-0.745062000	-5.302926000
H	-2.387571000	0.937702000	-4.606658000
H	0.984443000	-1.286973000	-6.105971000
C	-0.762415000	0.650326000	-3.233424000
C	1.134186000	-0.601687000	-4.076841000
H	-1.245800000	1.209625000	-2.432444000
H	2.130839000	-1.019381000	-3.934486000
C	0.520710000	0.099074000	-3.013195000
H	-1.285033000	-0.306147000	-6.472748000
N	1.151087000	0.231159000	-1.791368000
H	0.847313000	1.018131000	-1.225980000
H	2.162377000	0.141764000	-1.810223000
Bi	1.362331000	2.587037000	-4.784662000
Cl	0.744996000	3.863806000	-2.744023000
Cl	1.990478000	4.523020000	-6.204323000
Cl	3.648782000	1.968700000	-4.036713000

NO_2-C_6H_5···$BiCl_3$

C	-0.139158000	0.038321000	-5.570614000
C	-0.653309000	-1.069428000	-4.887330000
C	0.374358000	1.128556000	-4.859333000
H	-1.058202000	-1.914107000	-5.443126000
H	0.767225000	1.992772000	-5.393268000
C	-0.651263000	-1.096730000	-3.491400000
C	0.383656000	1.114773000	-3.463151000
H	-1.040895000	-1.942973000	-2.929768000
H	0.778032000	1.944305000	-2.880241000
C	-0.129221000	-0.001750000	-2.803948000
H	-0.155725000	0.060048000	-6.658986000
N	-0.120672000	-0.024719000	-1.317296000
O	-0.588684000	-1.020457000	-0.766131000
O	0.351897000	0.954273000	-0.740953000
Bi	-3.117193000	1.446445000	-4.432724000
Cl	-3.159369000	1.559208000	-6.898552000
Cl	-3.603755000	3.813178000	-3.964239000
Cl	-5.364030000	0.500045000	-4.090758000

OCHO-C_6H_5···$BiCl_3$

C	0.212640000	0.312851000	-5.637810000
C	-0.693400000	-0.655104000	-5.192424000
C	0.947591000	1.051578000	-4.706598000
H	-1.284871000	-1.221047000	-5.910765000
H	1.652513000	1.811643000	-5.041830000
C	-0.871640000	-0.895977000	-3.825870000
C	0.783753000	0.821346000	-3.338790000
H	-1.586134000	-1.638218000	-3.483203000
H	1.345058000	1.387924000	-2.597043000
C	-0.119211000	-0.153540000	-2.908033000
H	0.338118000	0.492302000	-6.704552000
O	-0.282909000	-0.255538000	-1.528758000
C	-0.483428000	-1.474896000	-0.930205000
O	-0.474981000	-2.548701000	-1.466925000
H	-0.639800000	-1.284902000	0.146407000
Bi	-2.531157000	1.957509000	-4.190530000
Cl	-3.834072000	0.835206000	-2.414406000
Cl	-3.881687000	1.248404000	-6.132736000
Cl	-3.458503000	4.233743000	-3.919792000

OH-C_6H_5···$BiCl_3$

C	-0.384894000	0.137484000	-5.660105000
C	-1.027593000	0.914078000	-4.691449000
C	0.560899000	-0.812405000	-5.258024000
H	-1.759699000	1.664612000	-4.986910000
H	1.067957000	-1.424785000	-6.003472000
C	-0.721954000	0.755714000	-3.335185000
C	0.875408000	-0.981828000	-3.909582000
H	-1.217187000	1.371481000	-2.583553000
H	1.617940000	-1.711090000	-3.588043000
C	0.234931000	-0.196004000	-2.938529000
H	-0.621711000	0.269110000	-6.714476000
O	0.588021000	-0.388365000	-1.640511000
H	0.335429000	0.395209000	-1.120679000
Bi	1.848143000	2.437628000	-4.158484000
Cl	1.656502000	2.872549000	-1.711720000
Cl	0.504133000	4.365129000	-4.919799000
Cl	4.099120000	3.418672000	-4.479263000

Cl···π arene adducts

C_2H_3-C_6H_5···$BiCl_3$

Bi	4.567631000	-0.232587000	0.819587000
C	-0.260465000	1.468953000	-0.097091000
C	-0.397841000	0.806589000	-1.321295000
C	-0.330329000	-0.586199000	-1.359692000
C	-0.132017000	-1.307436000	-0.183585000
C	-0.000326000	-0.658313000	1.056790000
C	-0.063568000	0.747786000	1.075589000
C	0.193622000	-1.467232000	2.263817000
C	0.305450000	-1.030513000	3.526424000
H	0.263058000	0.026727000	3.787988000
H	0.453703000	-1.729945000	4.347355000
H	0.254780000	-2.544189000	2.083079000

CF_3-C_6H_5···$BiCl_3$

C	0.069715000	0.296645000	-5.504524000
C	-0.947823000	-0.545004000	-5.056088000
C	1.013824000	0.793545000	-4.602846000
H	-1.689015000	-0.929086000	-5.756288000
H	1.799179000	1.464942000	-4.947467000
C	-1.027783000	-0.891810000	-3.706486000
C	0.940264000	0.451142000	-3.255338000
H	-1.823750000	-1.542791000	-3.349873000
H	1.664292000	0.851354000	-2.546827000
C	-0.082948000	-0.391848000	-2.808464000
H	0.122483000	0.579172000	-6.555537000
C	-0.138388000	-0.747762000	-1.347114000

H	0.048293000	1.281813000	2.018817000	F	-0.130328000	0.361983000	-0.554647000
H	-0.301283000	2.557785000	-0.060386000	F	-1.246862000	-1.461224000	-1.020502000
H	-0.549522000	1.375120000	-2.238703000	F	0.939692000	-1.496259000	-0.968402000
H	-0.428573000	-1.113728000	-2.308596000	Bi	-0.587500000	4.751979000	-4.026046000
H	-0.074469000	-2.396755000	-0.216416000	Cl	0.069046000	4.055363000	-6.290569000
Cl	3.561980000	-2.449325000	1.149955000	Cl	-1.993042000	2.844601000	-3.412392000
Cl	3.281679000	0.490164000	-1.134099000	Cl	1.472656000	4.143497000	-2.833760000
Cl	3.398815000	1.039781000	2.565116000				
$CHO\text{-}C_6H_5\cdots BiCl_3$				$Cl\text{-}C_6H_5\cdots BiCl_3$			
Bi	4.607969000	-0.020318000	-0.663727000	Bi	4.641267000	-0.797171000	-0.740894000
C	-0.012761000	1.329763000	0.038939000	C	0.009623000	1.426735000	0.259624000
C	0.068267000	0.497464000	-1.088371000	C	0.310714000	0.774980000	-0.937091000
C	-0.088406000	-0.887809000	-0.946362000	C	0.230127000	-0.615255000	-1.026539000
C	-0.333993000	-1.440818000	0.308698000	C	-0.157102000	-1.343790000	0.098416000
C	-0.417975000	-0.607340000	1.425891000	C	-0.458544000	-0.709146000	1.303646000
C	-0.254458000	0.776848000	1.291086000	C	-0.373000000	0.681908000	1.376382000
C	0.309982000	1.063265000	-2.433194000	Cl	-0.263626000	-3.080737000	-0.000754000
O	0.420922000	2.250377000	-2.684233000	H	-0.753691000	-1.300480000	2.168549000
H	0.384003000	0.296336000	-3.245559000	H	-0.605320000	1.182323000	2.316392000
H	-0.008988000	-1.525430000	-1.828964000	H	0.079579000	2.512000000	0.323449000
H	-0.453301000	-2.518198000	0.419897000	H	0.624161000	1.346493000	-1.809892000
H	-0.604663000	-1.037472000	2.410147000	H	0.472933000	-1.133382000	-1.952119000
H	-0.312905000	1.418455000	2.170164000	Cl	3.319645000	-2.733112000	-1.459537000
H	0.121033000	2.402920000	-0.096428000	Cl	3.772478000	0.864641000	-2.332725000
Cl	3.382129000	-1.176170000	-2.457141000	Cl	3.355801000	-0.160616000	1.244923000
Cl	3.622604000	2.207227000	-0.889717000				
Cl	3.311377000	-0.813934000	1.260965000				
$CN\text{-}C_6H_5\cdots BiCl_3$				$F\text{-}C_6H_5\cdots BiCl_3$			
Bi	4.633762000	-0.419905000	0.151762000	Bi	4.281916000	-0.312538000	1.592381000
C	-0.321839000	1.401621000	0.224602000	C	0.010904000	1.267773000	0.388884000
C	-0.742195000	0.899714000	-1.009238000	C	-0.213915000	1.128447000	-0.981986000
C	-0.626718000	-0.458916000	-1.288933000	C	-0.448599000	-0.131441000	-1.536503000
C	-0.084743000	-1.324746000	-0.323082000	C	-0.452840000	-1.234991000	-0.691094000
C	0.340282000	-0.818011000	0.917296000	C	-0.233428000	-1.126851000	0.676709000
C	0.218947000	0.542653000	1.183601000	C	0.000609000	0.140635000	1.212745000
C	0.037902000	-2.719100000	-0.606989000	F	-0.673673000	-2.466162000	-1.224377000
N	0.133647000	-3.856335000	-0.841595000	H	-0.230456000	-2.020747000	1.297930000
H	0.771224000	-1.494195000	1.653269000	H	0.189959000	0.241874000	2.280827000
H	0.561149000	0.934667000	2.140234000	H	0.207878000	2.250714000	0.814188000
H	-0.409533000	2.466838000	0.436869000	H	-0.201914000	2.004376000	-1.630307000
H	-1.159017000	1.570776000	-1.759564000	H	-0.621499000	-0.267133000	-2.602967000
H	-0.948037000	-0.860778000	-2.248472000	Cl	3.289632000	-0.507860000	-0.635912000
Cl	3.572628000	-2.609726000	0.421823000	Cl	3.282960000	1.816080000	2.308059000
Cl	3.059705000	0.582285000	-1.439834000	Cl	2.823463000	-1.853552000	2.832688000
Cl	3.826772000	0.641618000	2.220486000				
$NH_2\text{-}C_6H_5\cdots BiCl_3$				$NO_2\text{-}C_6H_5\cdots BiCl_3$			
C	-0.943512000	-0.410834000	-5.587983000	C	0.289028000	0.174785000	-5.439322000
C	-1.572662000	0.288805000	-4.555026000	C	0.028867000	-1.179091000	-5.211694000
C	0.363291000	-0.866560000	-5.396476000	C	0.249627000	1.087972000	-4.382897000
H	-2.591111000	0.655693000	-4.686626000	H	0.056912000	-1.888291000	-6.038048000
H	0.873472000	-1.410342000	-6.192210000	H	0.437280000	2.144953000	-4.565531000
C	-0.912409000	0.532017000	-3.353584000	C	-0.276301000	-1.627069000	-3.928444000
C	1.032274000	-0.628506000	-4.198785000	C	-0.052721000	0.655020000	-3.093961000
H	-1.411044000	1.085300000	-2.555914000	H	-0.491695000	-2.671645000	-3.716445000
H	2.056043000	-0.980970000	-4.063061000	H	-0.108151000	1.342874000	-2.254027000
C	0.403822000	0.077471000	-3.157217000	C	-0.312229000	-0.698343000	-2.890066000
H	-1.462743000	-0.596850000	-6.527149000	H	0.512244000	0.523280000	-6.447161000
N	1.052692000	0.279196000	-1.938679000	N	-0.657429000	-1.163006000	-1.527739000
H	0.725353000	1.089822000	-1.421807000	O	-0.896488000	-2.363355000	-1.377018000
H	2.066347000	0.277018000	-2.000917000	O	-0.687800000	-0.321539000	-0.627441000
Bi	2.421511000	3.915388000	-4.073128000	Bi	-3.765420000	2.633903000	-4.956822000
Cl	0.908498000	2.885855000	-5.682051000	Cl	-2.263030000	3.645601000	-3.297606000
Cl	0.969787000	3.906168000	-2.079560000	Cl	-2.250720000	2.646135000	-6.894119000

Cl	3.844508000	1.976906000	-3.533039000	Cl	-3.558225000	0.278092000	-4.321531000
OCHO-$C_6H_5\cdots BiCl_3$				OH-$C_6H_5\cdots BiCl_3$			
C	0.309952000	-0.243446000	-5.855289000	C	-0.659170000	-0.443817000	-5.825778000
C	-0.552649000	-1.118154000	-5.193991000	C	-0.966046000	0.677940000	-5.053914000
C	0.957569000	0.763403000	-5.136530000	C	0.223272000	-1.401268000	-5.317169000
H	-1.069523000	-1.899673000	-5.750504000	H	-1.651064000	1.436014000	-5.434451000
H	1.624642000	1.459631000	-5.644166000	H	0.470525000	-2.282704000	-5.909445000
C	-0.774306000	-1.006500000	-3.820024000	C	-0.401274000	0.844819000	-3.789629000
C	0.749769000	0.888369000	-3.764503000	C	0.800534000	-1.242490000	-4.058794000
H	-1.448901000	-1.685178000	-3.308474000	H	-0.650234000	1.719185000	-3.186440000
H	1.232255000	1.673514000	-3.184476000	H	1.495740000	-1.978914000	-3.657110000
C	-0.108021000	-0.001399000	-3.118033000	C	0.490439000	-0.113356000	-3.291492000
H	0.472172000	-0.341340000	-6.928175000	H	-1.102330000	-0.571425000	-6.812533000
O	-0.302049000	0.256307000	-1.754637000	O	1.086179000	0.004282000	-2.065442000
C	-0.562918000	-0.732846000	-0.856881000	H	0.875122000	0.881000000	-1.695875000
O	-0.617005000	-1.918383000	-1.061390000	Bi	1.878399000	4.466096000	-2.870852000
H	-0.710691000	-0.245804000	0.125023000	Cl	0.882734000	3.235495000	-0.963116000
Bi	-3.738661000	2.632172000	-3.176139000	Cl	2.977239000	2.599085000	-3.988956000
Cl	-2.854124000	1.838503000	-5.316297000	Cl	-0.127595000	4.634216000	-4.267109000
Cl	-3.826758000	0.494408000	-1.963051000				
Cl	-1.671905000	3.550570000	-2.224196000				
Bi···R adducts							
Cl-$C_6H_5\cdots BiCl_3$				CN-$C_6H_5\cdots BiCl_3$			
Bi	2.824072000	0.164567000	0.082603000	Bi	3.361443000	-0.363415000	-0.422124000
C	0.432223000	3.331293000	-4.385636000	C	2.229745000	5.390528000	3.454926000
C	0.536071000	1.956582000	-4.603954000	C	3.014445000	4.947188000	2.387130000
C	0.295744000	1.055691000	-3.565599000	C	2.722003000	3.743631000	1.753197000
C	-0.049675000	1.561602000	-2.315254000	C	1.628077000	2.981429000	2.202586000
C	-0.155885000	2.928151000	-2.070414000	C	0.836798000	3.423607000	3.277476000
C	0.087643000	3.812973000	-3.121627000	C	1.144017000	4.630310000	3.897990000
Cl	-0.346285000	0.437991000	-0.997557000	C	1.337954000	1.744898000	1.560445000
H	-0.411052000	3.289571000	-1.076615000	N	1.129036000	0.732237000	1.020928000
H	0.013222000	4.885214000	-2.943392000	H	-0.004337000	2.819625000	3.613762000
H	0.625860000	4.028310000	-5.200062000	H	0.535561000	4.978795000	4.731498000
H	0.812983000	1.575543000	-5.586357000	H	2.466310000	6.334190000	3.945878000
H	0.387298000	-0.017514000	-3.717237000	H	3.861044000	5.541247000	2.045518000
Cl	5.056992000	-0.186893000	1.085255000	H	3.324374000	3.381430000	0.921443000
Cl	2.884093000	2.620562000	-0.043483000	Cl	4.732309000	0.357284000	1.503455000
Cl	3.408302000	-0.343987000	-2.254033000	Cl	3.397216000	1.787514000	-1.642955000
				Cl	5.161524000	-1.499270000	-1.700155000
F-$C_6H_5\cdots BiCl_3$				NH_2-$C_6H_5\cdots BiCl_3$			
Bi	3.667067000	-0.133634000	0.280548000	C	0.967329000	-0.662072000	-0.809173000
C	0.555169000	5.062723000	-0.957027000	C	0.028327000	0.368387000	-0.888526000
C	1.132353000	4.280734000	-1.959465000	C	1.664929000	-1.042751000	-1.957394000
C	1.285819000	2.903672000	-1.783142000	H	-0.523984000	0.674393000	-0.000294000
C	0.846479000	2.352349000	-0.589738000	H	2.399954000	-1.845855000	-1.909279000
C	0.272677000	3.095279000	0.430166000	C	-0.214221000	1.013948000	-2.100171000
C	0.128040000	4.470327000	0.233250000	C	1.431399000	-0.404289000	-3.174518000
F	0.998821000	0.994393000	-0.402183000	H	-0.950032000	1.818083000	-2.156886000
H	-0.040826000	2.609438000	1.352268000	H	1.982482000	-0.699195000	-4.068919000
H	-0.316312000	5.078778000	1.020172000	C	0.482860000	0.626070000	-3.252998000
H	0.442437000	6.136365000	-1.101390000	H	1.153251000	-1.164266000	0.138988000
H	1.472830000	4.740559000	-2.886544000	N	0.314424000	1.338245000	-4.460926000
H	1.747661000	2.272730000	-2.540038000	H	-0.573515000	1.825923000	-4.559227000
Cl	5.778888000	-1.173466000	1.011962000	H	0.539804000	0.812738000	-5.304019000
Cl	3.785134000	1.904723000	1.648246000	Bi	2.336201000	3.231109000	-4.563098000
Cl	4.461637000	0.797840000	-1.856765000	Cl	4.115153000	4.930107000	-4.977404000
				Cl	0.528463000	4.455091000	-5.737958000
				Cl	3.031972000	1.669730000	-6.365115000
NO_2-$C_6H_5\cdots BiCl_3$				OH-$C_6H_5\cdots BiCl_3$			
C	-0.765205000	-4.967212000	-1.701495000	C	-4.202764000	1.000327000	-2.542260000
C	-2.140133000	-4.730496000	-1.791895000	C	-3.176804000	0.617933000	-1.676575000

C	0.145685000	-3.913133000	-1.820704000	C	-3.926501000	1.185292000	-3.898843000
H	-2.845190000	-5.555617000	-1.702735000	H	-3.378943000	0.475576000	-0.615344000
H	1.215781000	-4.103652000	-1.753694000	H	-4.720951000	1.483361000	-4.582625000
C	-2.615325000	-3.438871000	-2.000220000	C	-1.881634000	0.422190000	-2.156741000
C	-0.311055000	-2.614924000	-2.028971000	C	-2.637735000	0.988706000	-4.392980000
H	-3.677474000	-3.221348000	-2.081657000	H	-1.073791000	0.140291000	-1.479380000
H	0.370204000	-1.773732000	-2.131361000	H	-2.411302000	1.121396000	-5.450570000
C	-1.687650000	-2.402367000	-2.112872000	C	-1.622471000	0.606786000	-3.515623000
H	-0.400698000	-5.981267000	-1.539741000	H	-5.211322000	1.154952000	-2.161733000
N	-2.176453000	-1.039965000	-2.336849000	O	-0.353641000	0.435110000	-4.044117000
O	-3.403741000	-0.868101000	-2.408250000	H	0.252730000	0.114704000	-3.345739000
O	-1.353297000	-0.128271000	-2.459175000	Bi	1.413735000	2.647382000	-4.361822000
Bi	-3.599468000	1.552089000	-4.080551000	Cl	2.432250000	1.025391000	-2.763651000
Cl	-2.697934000	-0.069787000	-5.714515000	Cl	-0.018332000	3.844555000	-2.771736000
Cl	-3.573919000	3.552790000	-5.552227000	Cl	3.314691000	4.230249000	-4.392690000
Cl	-5.989789000	0.983286000	-4.295410000				
$BiCl_3$ interaction with NO_2CH_3							
Motif A				Motif B			
C	1.090330000	3.796431000	2.789556000	C	0.962669000	1.069615000	6.439314000
H	0.096959000	3.548761000	3.167948000	H	-0.042788000	0.758971000	6.724239000
H	1.176507000	4.826287000	2.437689000	H	1.711749000	0.283919000	6.586522000
H	1.389876000	3.095543000	1.999987000	H	1.018821000	1.413639000	5.399649000
N	2.077206000	3.591790000	3.889076000	N	1.354452000	2.225174000	7.300397000
O	1.816235000	2.751775000	4.748815000	O	0.542173000	2.673874000	8.091556000
O	3.131833000	4.230285000	3.832017000	O	2.506414000	2.674351000	7.155924000
Bi	4.929213000	1.918311000	4.681219000	Bi	4.653051000	2.222312000	5.346501000
Cl	7.016782000	2.887627000	3.804429000	Cl	6.549989000	1.960651000	3.785320000
Cl	5.817482000	-0.259637000	5.441999000	Cl	4.371227000	-0.174097000	5.890106000
Cl	3.977346000	1.144837000	2.524095000	Cl	2.884214000	2.422861000	3.627602000
NO_2CH_3							
C	1.028604000	4.012105000	2.834891000				
H	0.321674000	4.389442000	3.579558000				
H	1.438666000	4.809885000	2.214124000				
H	0.545078000	3.234714000	2.235402000				
N	2.156588000	3.356478000	3.582528000				
O	1.847713000	2.455406000	4.358490000				
O	3.295859000	3.761339000	3.365218000				
NO_2-C_6H_5 with explicit NO_2CH_3 molecules							
C	-0.721354000	0.729646000	-6.646500000				
C	-1.222314000	-0.536120000	-6.294825000				
C	-0.057184000	1.519705000	-5.694051000				
H	-1.756384000	-1.152975000	-7.028627000				
H	0.368989000	2.497641000	-5.960421000				
C	-1.051807000	-1.035370000	-4.997454000				
C	0.093698000	1.050715000	-4.384942000				
H	-1.453018000	-2.011914000	-4.697672000				
H	0.600843000	1.652069000	-3.622537000				
C	-0.393653000	-0.223294000	-4.066263000				
H	-0.877211000	1.105463000	-7.667177000				
N	-0.172431000	-0.713149000	-2.701582000				
O	-0.011207000	-1.924490000	-2.532576000				
O	-0.129139000	0.131636000	-1.809639000				
C	2.078235000	-2.985655000	-4.703086000				
H	2.972676000	-2.451853000	-4.342023000				
H	1.220652000	-2.757832000	-4.034311000				
H	2.220133000	-4.077575000	-4.770170000				
N	1.699980000	-2.493853000	-6.055627000				
O	2.122146000	-1.394283000	-6.418118000				
O	0.949753000	-3.205133000	-6.727426000				
C	2.802385000	-0.888412000	-0.440829000				
H	3.406564000	-1.590229000	0.155596000				
H	3.025165000	0.172276000	-0.206226000				
H	1.718934000	-1.084017000	-0.305103000				

N	3.126274000	-1.118613000	-1.876816000	
O	3.794803000	-2.106346000	-2.175270000	
O	2.681996000	-0.311687000	-2.702771000	
C	-2.909238000	3.004400000	-4.236907000	
H	-3.285060000	2.273192000	-4.974062000	
H	-3.700286000	3.737414000	-3.987479000	
H	-2.004125000	3.529509000	-4.583245000	
N	-2.571591000	2.301387000	-2.955388000	
O	-3.181631000	1.261994000	-2.701645000	
O	-1.742871000	2.829583000	-2.221359000	
C	-2.153708000	-0.007115000	0.444375000	
H	-2.662164000	0.373469000	1.349336000	
H	-2.125521000	0.781089000	-0.329971000	
H	-2.626771000	-0.921766000	0.051812000	
N	-0.737283000	-0.306294000	0.823368000	
O	-0.352251000	-1.470183000	0.739651000	
O	-0.041363000	0.640948000	1.188976000	
C	0.962718000	-1.314624000	-9.550513000	
H	1.235617000	-2.237329000	-10.091341000	
H	1.425261000	-0.405907000	-9.967481000	
H	1.234153000	-1.425604000	-8.478843000	
N	-0.520057000	-1.185159000	-9.602753000	
O	-1.190632000	-2.187903000	-9.370198000	
O	-1.004208000	-0.072362000	-9.837291000	
C	3.688271000	1.570319000	-5.296945000	
H	4.779292000	1.409361000	-5.301337000	
H	3.167680000	0.607692000	-5.125548000	
H	3.314171000	2.049093000	-6.218640000	
N	3.358380000	2.443466000	-4.125714000	
O	4.088540000	2.375874000	-3.142893000	
O	2.342832000	3.140081000	-4.205740000	
C	-4.757692000	-1.350189000	-3.692178000	
H	-4.485139000	-0.322715000	-3.995286000	
H	-4.365374000	-2.068944000	-4.431135000	
H	-5.846152000	-1.437414000	-3.540780000	
N	-4.080066000	-1.581632000	-2.378771000	
O	-4.726312000	-1.361922000	-1.358077000	
O	-2.900608000	-1.932452000	-2.409148000	
C	1.599927000	2.642743000	-0.977164000	
H	0.920725000	2.289918000	-0.177333000	
H	1.170755000	3.486151000	-1.539339000	
H	1.850786000	1.790391000	-1.630133000	
N	2.865080000	3.099110000	-0.322404000	
O	3.607176000	2.225201000	0.136543000	
O	3.074620000	4.303049000	-0.248795000	
C	0.616099000	2.857387000	-9.663929000	
H	0.092188000	1.912874000	-9.900584000	
H	-0.046886000	3.601000000	-9.193022000	
H	1.086775000	3.265998000	-10.577731000	
N	1.727386000	2.536546000	-8.708269000	
O	2.425893000	1.558957000	-8.970840000	
O	1.879995000	3.278489000	-7.740641000	
C	-1.935419000	-4.629010000	-7.402598000	
H	-1.111543000	-4.034749000	-7.842562000	
H	-1.690918000	-4.892926000	-6.360375000	
H	-2.166067000	-5.505238000	-8.030425000	
N	-3.132279000	-3.730926000	-7.396362000	
O	-3.884124000	-3.767460000	-8.362254000	
O	-3.254476000	-2.967205000	-6.432737000	
C	-4.200557000	-0.291163000	-8.416145000	
H	-5.055176000	0.021281000	-9.045514000	
H	-3.281159000	-0.329836000	-9.028858000	
H	-4.402680000	-1.251499000	-7.912633000	
N	-4.016737000	0.772741000	-7.377669000	

O	-4.427138000	0.537365000	-6.240683000	
O	-3.479550000	1.821097000	-7.727541000	
